# Problem-solving in virtual environment simulations prior to direct instruction for differential diagnosis in medical education: An experimental study

**DOI:** 10.12688/mep.19348.1

**Published:** 2022-10-19

**Authors:** Christian Fässler, Tanmay Sinha, Christian Marc Schmied, Jörg Goldhahn, Manu Kapur

**Affiliations:** 1 Department of Humanities, Social and Political Sciences, ETH Zürich, Zürich, 8092, Switzerland; 2 Department of Health Sciences and Technology, ETH Zürich, Zürich, 8092, Switzerland; 3 University Heart Center, University Hospital Zürich, Zürich, 8091, Switzerland

**Keywords:** Clinical knowledge, clinical reasoning skills, learning activity sequencing, problem-solving prior to instruction, situated learning, transfer

## Abstract

**Background:** Despite acquiring vast content knowledge about the functioning of the human body through university teaching, medical students struggle to transfer that knowledge to one of the core disciplinary practices – differential diagnosis (DD). The authors aimed to overcome this problem by implementing computer-based virtual environment (CVE) simulations in medical education courses.

**Methods: **In an experimental study, the authors compared problem-solving in medical CVEs prior to instruction with an instruction-first approach. They compared the effects on isomorphic testing and transfer performance of clinical knowledge (CK) and clinical reasoning skills (CRS) as well as evoked learning mechanisms. The study took place in spring 2021 with undergraduate medical students in the scope of a medical trajectory course. Due to COVID-19 issues participants completed all study activities remotely from home.

**Results:** The authors did not find any learning activity sequence to be superior to the other. However, when looking at the two learning activities individually, they found that problem-solving in CVEs as well as direct instruction are equally effective at imparting content knowledge, whereas problem-solving in CVE with formative feedback imparts clinical reasoning skills better than mere instruction.

**Conclusions:** The findings indicate that only the problem-solving learning activity in CVEs imparts CRS and that such skills cannot be taught by theoretical instruction only. The present study has a high level of ecological validity because it took place in a realistic setting where students had to perform all learning and testing tasks autonomously.

## Introduction

Empirical studies reveal that undergraduate medical students struggle on counseling patients in the clinical practice of medicine ( Prince *et al.*, 2005). A major reason for the students’ struggling in practice might be that CK which was acquired in a university setting does not transfer to clinical practice ( Collard *et al.*, 2016). We specify *CK* as declarative and conceptual knowledge about specific diseases and the process of DD. Yet, we identify the failure to transfer in current methods of instruction where the focus is on first providing direct instruction on basic knowledge without adequate attention to situate this knowledge in disciplinary practice. In the present study, we also focused on the underlying mental process of DD - clinical reasoning. *Clinical reasoning* describes the thinking and decision-making process associated with clinical practice ( Higgs & Jones, 2008). Clinical reasoning must be applied in every moment of patient attendance and is fundamental for a timely diagnosis of diseases. We annotate that in this study we focused on the conceptual aspects of DD (e.g., why certain procedures work), hence CRS, and not on the execution of procedures (e.g., auscultate the heart during the physical examination). Accordingly, to overcome the issue of transfer, we proposed a transition to situations as starting points (SSP) ( Michaud & Jucker-Kupper, 2017) where (a) the acquisition of CK and CRS must be situated in disciplinary practice and (b) learning must be anchored to an actual disciplinary problem. With our study, we aimed to enhance isomorphic testing and near and far transfer outcomes of CK and CRS through CVEs in university teaching through aspects of situated learning. Inter alia, the ability to transfer is important because medical students cannot be confronted with all possible situations in their medical studies they will face later in their professional career as doctors.

For the purpose of the present work, we define *transfer* as acquiring CK and CRS in one context and applying it to another. Relating thereto we define the following modalities: (a) *near transfer* which means to transfer CK and CRS acquired in learning activities based on one SSP, to an assessment method with content building on the *same* SSP but a *different* diagnosis and (b) *far transfer* which means to transfer CK and CRS acquired in learning activities based on one SSP, to an assessment method with content building on *another* SSP. Furthermore, *isomorphic* means to acquire CK and CRS in learning activities based on one SSP and employ it in an assessment method with content building on the *same* SSP and the *same* diagnosis.

Whilst we acknowledge that there might be several options for implementing situated learning and SSPs and to enhance transfer, we aimed to explore the use of medical CVE simulations. Such virtual patient scenarios allow medical students to learn and experience in safe environments from which transfer to other simulated situations or clinical practice can be enhanced. Furthermore, CVE simulations offer a unique possibility to generate almost any relevant medical scenario and align such scenarios with the SSP approach. Additionally, CVE tools and platforms allow individualized learning. Because CVE simulations are scalable and not limited to a certain number of students, their use in medical education is also potentially beneficial for institutions and lecturers from an economic and efficiency perspective in the long-term. However, in empirical research it has yet to be shown how and when CVE simulations are effective in medical education and enhancing transfer.

Recent meta-analytic evidence from the learning sciences suggests that *problem-solving preceding instruction*, on average, results in better conceptual understanding and transfer outcomes than instruction-first learning approaches for comparisons carried out in the domain of medicine ( Sinha & Kapur, 2021a). However, there also are limitations on this approach where the *instruction preceding problem-solving* sequence might be more appropriate. This is why we compared the problem-solving prior to instruction (CVE-I) with the instruction-first (I-CVE) learning activity sequence for DD education and their effect on CK and CRS acquisition and transfer. In our study, problem-solving was represented by a patient scenario in CVE, which situates learning in disciplinary practice. In such instructional designs, the first learning activity is assumed to trigger mechanisms which prepare students to benefit from the subsequent learning activity ( Loibl *et al.*, 2017). Consequently, we operationalized the following thereto related learning mechanisms: knowledge gap awareness, state curiosity, germane cognitive load, positive affect and negative affect ( Sinha *et al.*, 2020). By the varied sequencing of the two learning activities and the assessment of triggered learning mechanisms we aimed to evaluate when and how (a) the acquisition and transfer of CK an CRS through can be best enhanced and (b) CVEs are effective in fostering the acquisition and transfer of CK an CRS.

## Methods

### Participants

The present study took place in scope of a third-year medical course at an open admission highly ranked university in Western Europe. Students were randomly assigned to the intervention groups before the course start. We could recruit *N* = 61 (63.93% female, *n* = 39) students who gave written (online) informed consent to include their data in the study whereof *n* = 34 (58.82% female, *n* = 20) students were assigned to the CVE-I group and *n* = 27 (70.37% female, *n* = 19) to the I-CVE group. No compensation was given to study participants. This study was approved by the Ethics Committee of ETH Zurich.

### Study context

Due to COVID-19 ( World Health Organization (WHO), 2020) issues, the study took place online. The students completed all activities of the study autonomously from home on I-Human Patients, Moodle, and Qualtrics. However, we sent detailed instructions to students via e-mail about the proceeding of the next study phase or day.

### Experimental design

There were two subject groups going either through the *CVE-I* or *I-CVE* learning activity sequence in the intervention phase. The pre-intervention phase took place three to five days prior to the intervention. The intervention phase took place within one morning where participants followed their learning activities and intermediate testing according to their assigned sequence. The post-intervention phase took place over two days due to time considerations. In the afternoon of the intervention day, participants went through the isomorphic assessment. On the next day, students first went through the near transfer assessment and then through the far transfer assessment. Please refer to Extended Data 1 ( Fässler, 2022f) for an illustration of the experimental design.

### Learning materials and measures

***CVE platform*.** We used the interactive learning platform I-Human Patients for our medical CVE simulations. Please refer to Extended Data 2 ( Fässler, 2022g) for an illustration of the platform. To assess and quantify CRS we extracted the following measures from the CVE scenarios:

- *History*: asking correct questions when obtaining the patient’s history
- *History Relevance*: correctly asked questions in relation to extraneous and missed ones
- *Physical Examination*: selecting correct physical examinations during the patient encounter based on findings in the patient history (or electronic patient record)
- *Physical Examination Relevance*: correctly selected examination in relation to extraneous and missed ones
- *Differential Diagnoses Selection*: selecting correct eligible differential diagnoses based on findings in the patient history and physical examination
- *Tests*: selecting correct clinical tests to include/exclude eligible differential diagnoses
- *Tests Relevance*: correctly selected clinical tests in relation to extraneous and missed clinical ones
- *Time*: time used to solve the CVE scenario

***Pre-intervention phase*.** In this phase, students had to solve a multiple-choice (MC) quiz with questions related to the medical topics covered in the intervention and post-intervention phase. This pre-intervention quiz was to assess students’ prior knowledge and to establish the baseline for potential CK acquisition and transfer caused by the intervention. Consequently, all questions of the pre-intervention quiz were asked again later in the study process when assessing the corresponding modality. We took quiz questions from the I-Human Patients platform because they are properly matched to the content of the CVE scenarios. MC questions were set up and answered in Moodle. We assessed CK in four modalities with MC questions: (a) intermediate, (b) isomorphic, (c) near transfer, and (d) far transfer. Please refer to the sections Intermediate Testing and Post-intervention Testing for question examples. Furthermore, please refer to Extended Data 3 ( Fässler, 2022h) for all implemented questions. Scores of all MC quizzes were transformed into percentage values.

***Problem-solving phase*.** In this phase, students worked independently through the CVE problem-solving scenario. The simulated DD process consisted of four stages: (a) taking the patient history by asking questions to the patient, (b) performing the physical examination by selecting appropriate examinations based on the findings in the history part, (c) selecting eligible differential diagnoses based on findings in the patient history and physical examination, and (d) selecting appropriate clinical tests to include/exclude eligible differential diagnoses. In the problem-solving scenario, individualized instant formative feedback was provided after each of these four stages. The problem-solving scenario was based on a simulated patient with the SSP headache where the DD process ended with migraine.

***Instruction phase*.** This phase was represented by a monologue video lecture of 25 minutes duration. This lecture consisted of (a) a theoretical introduction where a doctor provided information about headaches and the corresponding diagnostic approach and (b) a case review where the doctor worked through the DD process on an actual patient with migraine. The video lecture was provided via the I-Human Patients platform too.

***Intermediate testing*.** After the first learning activity, we collected students’ self-reported learning mechanisms measurements on a five-point Likert scale via questionnaires in Qualtrics: *germane cognitive load* ( Leppink *et al.*, 2014; 6 items, e.g., “This activity improved my understanding of the content that was covered *”*), *knowledge gap awareness* ( Glogger-Frey *et al.*, 2017; 5 items, e.g., “My knowledge was insufficient to carry out these tasks”), *state curiosity* ( Naylor, 1981; 9 items, e.g., “I feel like asking questions about what is happening”), *positive affect* ( Watson *et al.*, 1988; 10 items, e.g., determined, enthusiastic) and *negative affect* ( Watson *et al.*, 1988; 10 items, e.g., upset, distressed). Please refer to Extended Data 4 ( Fässler, 2022i) for all questionnaire items. We also introduced (a) an *intermediate* MC quiz on Moodle (9 items, e.g., “What is the difference between a primary and secondary headache?”) and (b) a CVE testing scenario on I-Human Patients which was shorter than the standard scenarios. Like the problem-solving scenario, the intermediate scenario was based on the SSP headache leading to migraine. However, the patient looked differently and the patient history and physical exam were replaced by an electronic patient record from which students had to extract the most relevant information to continue with the DD process. No feedback was provided.

***Post-intervention testing*.** This phase was split into three sections: isomorphic, near transfer and far transfer testing. Each of these three sections consisted of a CVE scenario and a MC quiz. During the isomorphic testing section, participants worked through the same scenario as in problem-solving scenario. However, the patient looked different and no feedback was provided. This was followed by an isomorphic MC quiz with questions about migraine (10 questions, e.g., “Your patient describes her/his headache as preceded by an aurea, diplopia, and loss of coordination. What is the most likely diagnosis?”). During the near transfer testing section, participants worked through a patient scenario which was also based on the SSP headache but led to subarachnoid hemorrhage. This was followed by a near transfer MC quiz with questions about subarachnoid hemorrhage (4 questions, e.g., “You suspect that your patient has a subarachnoid hemorrhage, but the non-contrast CT is negative. Which test would be best to perform next?”). During the far transfer testing section, participants worked through a patient scenario which was based on the SSP chest pain and lead to pulmonary embolism. This was followed by a far transfer MC quiz with questions about pulmonary embolism (6 questions, e.g., “Which of the following organ systems should be considered when investigating the cause of dyspnea?”).

### Analysis plan

For all statistical analyses we used the IBM SPSS, version 27 (IBM Corporation, Armonk, New York), and JASP, version 0.15 (Department of Psychological Methods, University of Amsterdam, Amsterdam), computer software. Before carrying out the analyses, we checked whether randomization of students to groups was effective. We did so by applying a Mann-Whitney U test on pre-intervention quiz scores for each CK modality. As alternative complementary analyses for null hypothesis significance testing, we used Bayesian informative hypotheses evaluation and corresponding non-parametric approaches in all analyses ( Hoijtink *et al.*, 2019). This allowed direct evaluation of the Bayes factor of a null hypothesis ( *BF _01_*) at hand or equivalence testing versus its alternative hypothesis ( *BF _10_*) for measure differences.

### Intermediate testing

***Learning mechanisms*.** We conducted an ANCOVA, controlling for average pre-intervention CK, for the comparison of feedback response. Furthermore, we conducted a MANCOVA, boot-strapped with 1000 replications, to compare the extent to which the posited learning mechanisms were triggered among the CVE-I and I-CVE groups. Please refer to for details about covariates included in the analyses.

***Clinical knowledge*.** We ran individual repeated measures ANOVAs, boot-strapped with 1000 replications, on pre-, intermediate-, and post-intervention quiz scores in all CK modalities. This was done to evaluate effects on CK improvement caused by the intervention. Subsequently, we ran individual ANCOVAs on post- intervention quiz scores, controlling for pre-intervention CK and problem-solving CRS scores, to compare the performance after the intervention for each CK modality between groups. The ANCOVA on intermediate quiz scores (after the respective first learning activity) controlled for intermediate pre-intervention CK only. Notably, these analyses were performed for declarative and conceptual knowledge questions separately. This was because the intervention might influence these two kinds of knowledge differently.

***Clinical reasoning skills*.** First, we ran a MANCOVA, boot-strapped with 1000 replications, on problem-solving scenario CRS scores, controlling for Time used to solve this scenario and overall pre-intervention CK. Second, we ran individual MANCOVAs, boot-strapped with 1000 replications, on the isomorphic, near transfer and far transfer scenario, controlling for Time used to solve the respective scenario, overall pre-intervention CK in the corresponding modality, problem-solving CRS scores, and feedback response to compare the performance in each CVE scenario among groups. The MANCOVA on intermediate scenario scores controlled for Time and overall intermediate pre-intervention quiz score only. To get a complete overview, we averaged the scores of all CRS factors in each scenario (problem-solving, intermediate, isomorphic, near transfer, and far transfer). We then ran ANCOVAs, boot-strapped with 1000 replications, on the averaged score of each scenario, controlling for Time in the respective scenarios, overall pre-intervention quiz score in the respective modality and mean problem-solving CRS score. The ANCOVA on intermediate CRS scores controlled for overall intermediate pre-intervention quiz score only.

## Results

Some students did not work through all study activities. Hence, datasets for these students were incomplete. However, we did not exclude any student from the analysis because multiple imputation did not have a significant influence on the results. Consequently, we ran the analyses with all students and the corresponding available data. Nevertheless, the number of students ( *N*) included varied among analyses.

### Pre-intervention testing

The Mann-Whitney U test on average pre-intervention quiz score did not reveal a significant difference between groups. Bayesian Mann-Whitney U testing confirmed this finding, *BF _01_* = 2.80. When considering prior CK in all modalities individually, no significant difference between groups was revealed in neither of the CK modalities. Bayesian Mann-Whitney U testing confirmed this finding, intermediate CK, *BF _01_* = 2.81; isomorphic CK, *BF _01_* = 3.45; near transfer CK, *BF _01_* = 2.07; far transfer CK, *BF _01_* = 1.71. Consequently, this is anecdotal to moderate evidence that groups were equal and randomization was effective. Please refer to Extended Data 5 ( Fässler, 2022j) for descriptives of prior knowledge and all other measures included in the present study.

### Problem-solving phase

**Figure 1.**
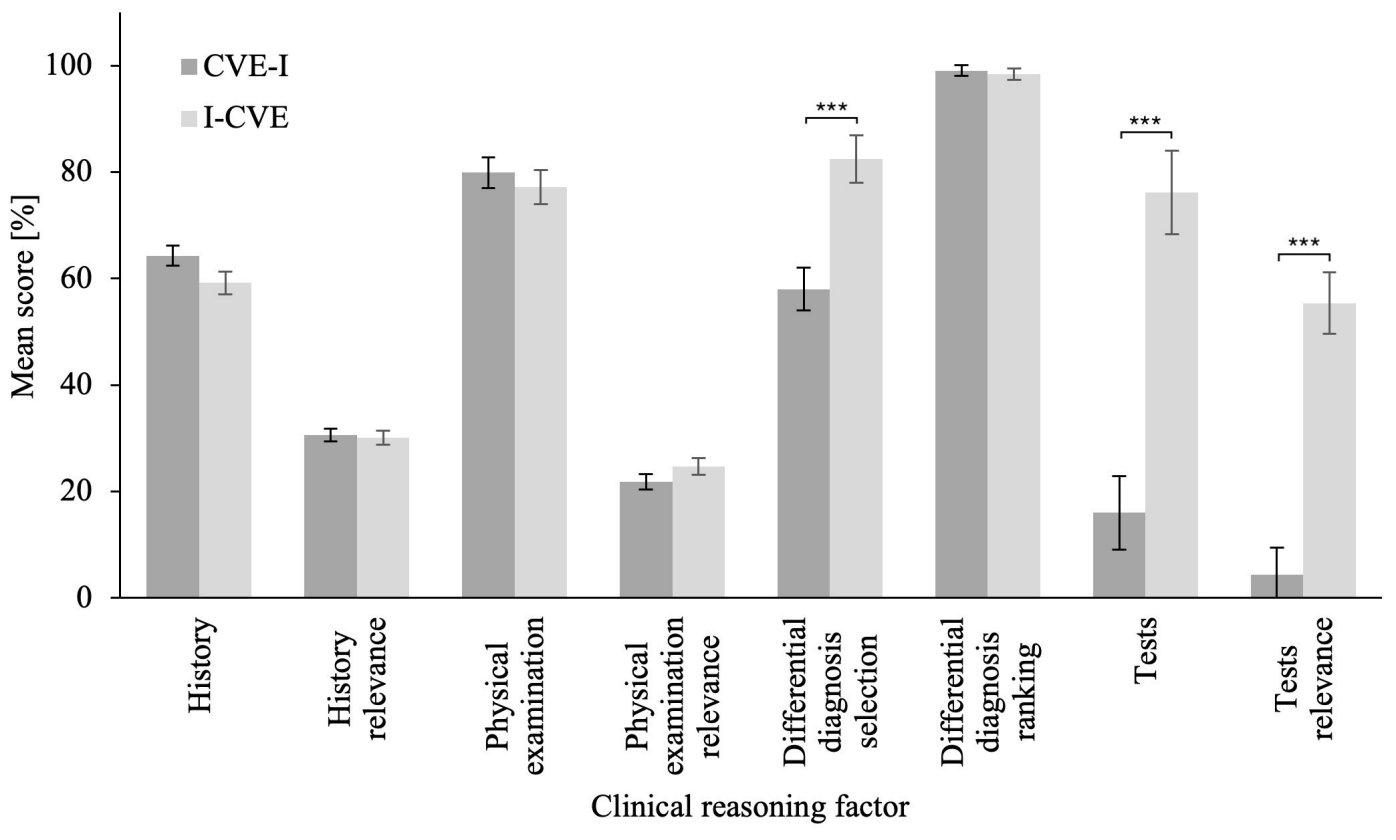
Clinical Reasoning Skills Scores in the Problem-Solving Scenario. The problem-solving phase CRS compares the scores of the first learning activity for the CVE-I group and of the second learning activity for the I-CVE group. Error bars represent standard error. * p ≤ .05, ** p ≤ .01, *** p ≤ .001.

**Figure 2.**
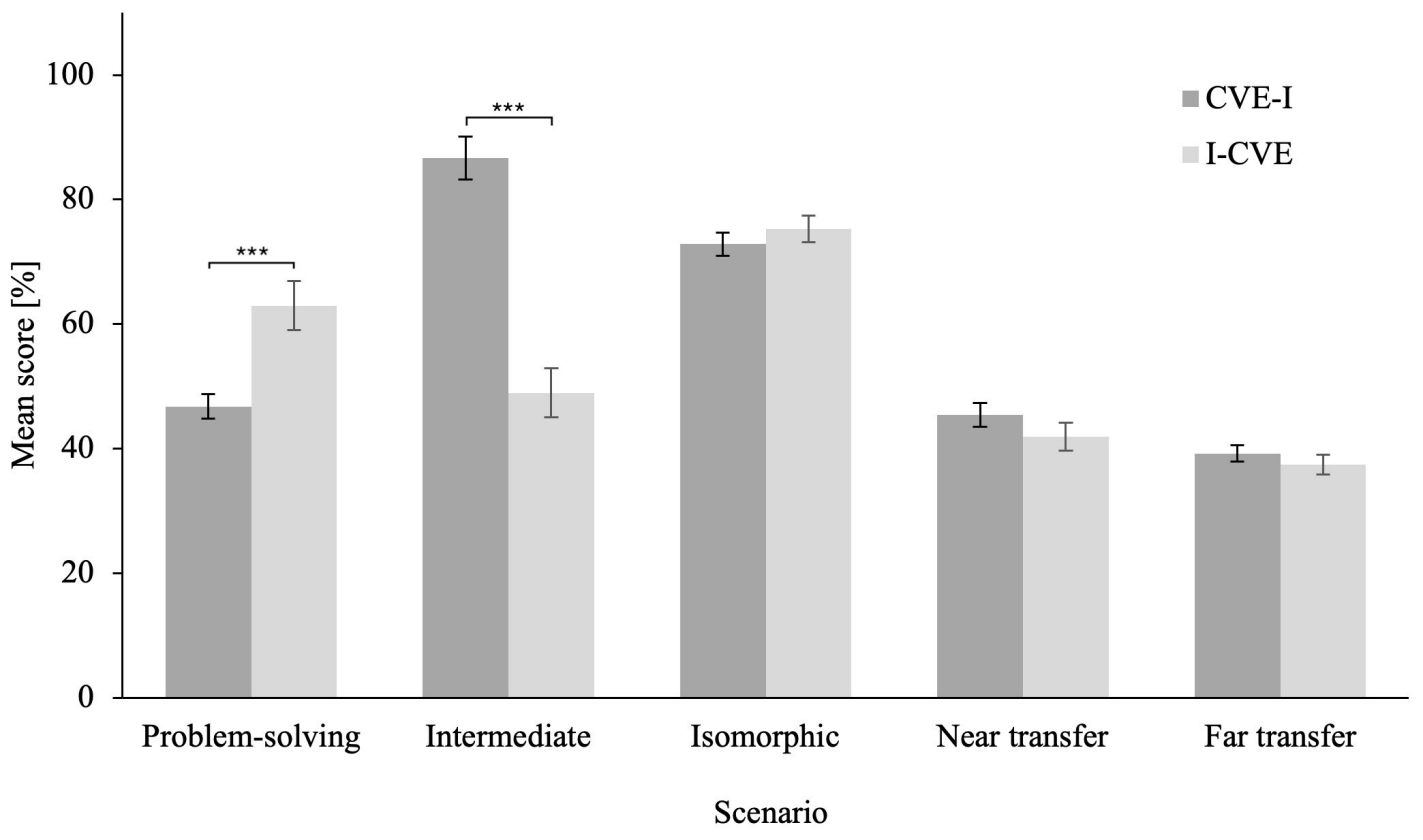
Averaged Score of Clinical Reasoning Skills Factors for Each Scenario. Error bars represent standard error. * p ≤ .05, ** p ≤ .01, *** p ≤ .001.

The MANCOVA on the problem-solving scenario revealed a significant difference in CRS scores between groups, *Wilk’s λ* = .463, *F*(8,48) = 6.97, *p* < .001, *η _p_²* = .537 ( Fässler, 2022e). Related univariate ANCOVAs revealed that the I-CVE group significantly outperformed the CVE-I group in Differential Diagnoses Selection, *F*(1,55) = 19.60, *p* < .001, *η _p_²* = .263, *BF _10_* = 35.35, Δ *M* = 24.46, 95% CI [12.21, 36.70]; Tests, *F*(1,55) = 30.98, *p* < .001, *η _p_²* = .360, *BF _10_* > 100, Δ *M* = 60.14, 95% CI [38.69, 81.59]; and Tests Relevance, *F*(1,55) = 42.16, *p* < .001, *η _p_²* = .434, *BF _10_* > 100, Δ *M* = 51.03, 95% CI [35.16, 66.90]. Please refer to Figure 1 for an illustration of the problem-solving scenario score. The ANCOVA on averaged CRS score revealed a that the I-CVE group significantly outperformed the CVE-I group, F(1,57) = 29.11, p < .001, *η _p_²* = .338 *, BF _10_* > 100, ΔM = 16.18, CI [10.18, 22.19]. Please refer to Figure 2 for an illustration of the ANCOVA results for all CVE scenarios. Furthermore, please refer to Extended Data 6 (( Fässler, 2022k) for descriptives of the ANCOVAs on all CVE scenarios.

### Intermediate testing

**Figure 3.**
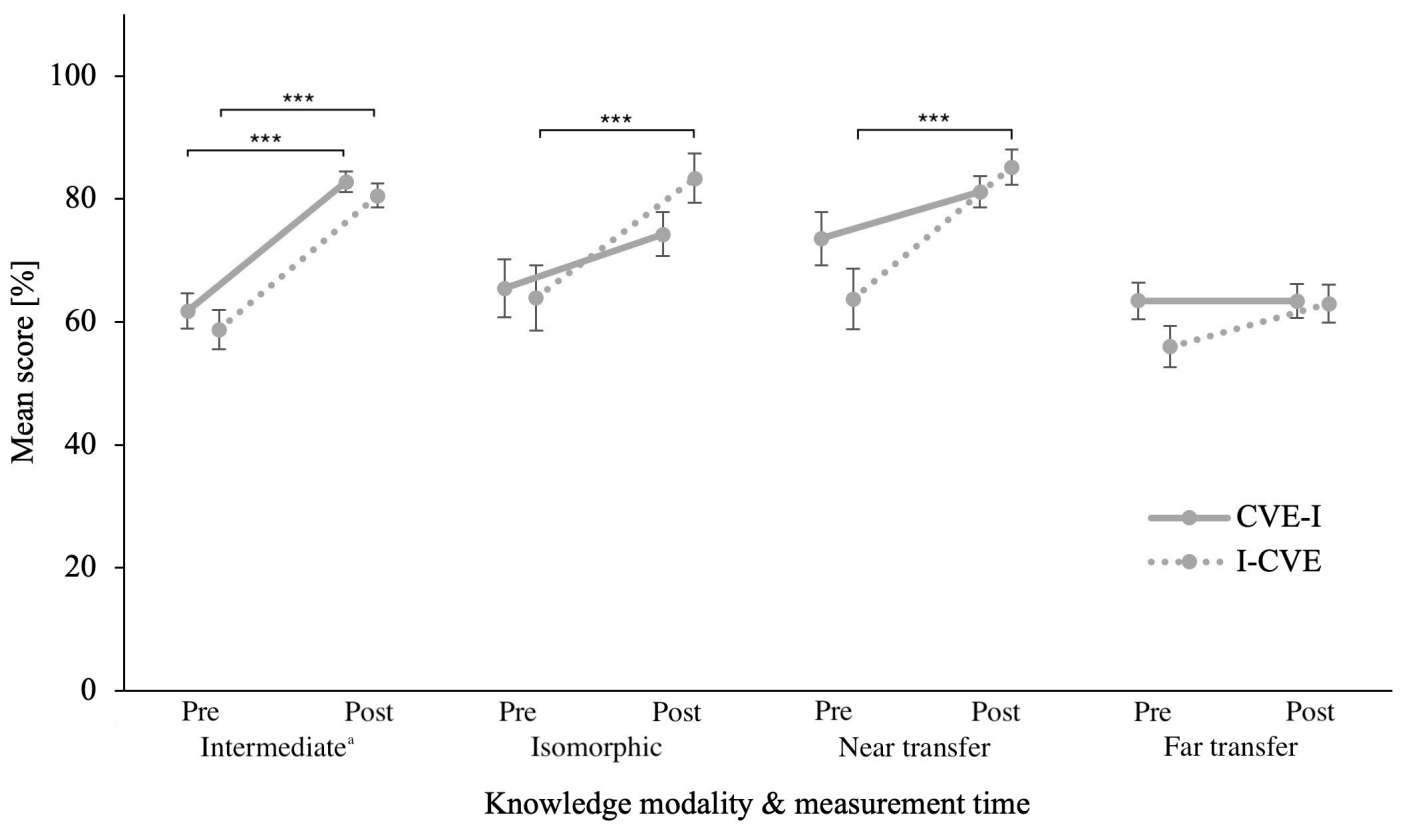
Pre- and Post- intervention Quiz Scores of Declarative Knowledge in all Modalities. Error bars represent standard error. * p ≤ .05, ** p ≤ .01, *** p ≤ .001 ^a^ Intermediate testing clinical knowledge was assesses after the respective first learning activity during the intervention phase.

Within-subjects effect analysis of the repeated measures ANOVA on intermediate *declarative* CK, revealed a significant difference between the pre-intervention and post-first learning activity quiz scores, *F*(1,59) = 88.51, *p* < .001, *η _p_²* = .600. However, between-subjects analysis revealed no significant effects of groups ( Fässler, 2022d). Post-hoc paired samples t-tests using Holm correction, revealed that both groups performed significantly better in the quiz after the first learning activity than in the pre-intervention quiz, CVE-I, *ΔM* = 21.03, 95% CI [12.75, 29.31], *t* = 6.95, *p* < .001; I-CVE *ΔM* = 21.85, 95% CI [12.56, 31.14], *t* = 6.42, *p* < .001. Corresponding *BF _10_* in this overall trend was >100 for pre-post comparison and 0.28 for group comparison. The ANCOVA on post-first learning activity quiz score did not reveal a significant difference between groups, *BF _01_* = 2.78. Please refer to Figure 3 for an illustration for post-first learning activity and post-intervention quiz scores for declarative knowledge in all quizzes when controlling for the covariates listed in Table 1.

**Table 1.** Analysis Plan Summary for ANCOVAs and MANCOVAs Included in the Present Work.

Measure	Dependent variables	Covariates			
		Pre intervention CK	Problem- solving CRS	Feedback response	Time ^a^
Learning mechanisms	Germane cognitive load, knowledge gap awareness, state curiosity, positive affect, negative affect	✓			
Clinical knowledge					
Intermediate	Intermediate CK quiz score	✓			
Isomorphic	Post-intervention isomorphic CK quiz score	✓	✓	✓	
Near transfer	Post-intervention near transfer CK quiz score	✓	✓	✓	
Far transfer	Post-intervention far transfer CK quiz score	✓	✓	✓	
Clinical reasoning skills					
Intermediate	History, History Relevance, Physical Examination, Physical Examination Relevance, Differential Diagnoses Selection, Differential Diagnosis Ranking Tests, Tests Relevance	✓			✓
Isomorphic		✓	✓	✓	✓
Near Transfer		✓	✓	✓	✓
Far Transfer		✓	✓	✓	✓

^a ^Time used to solve the CVE patient problem in the respective scenario

**Figure 4.**
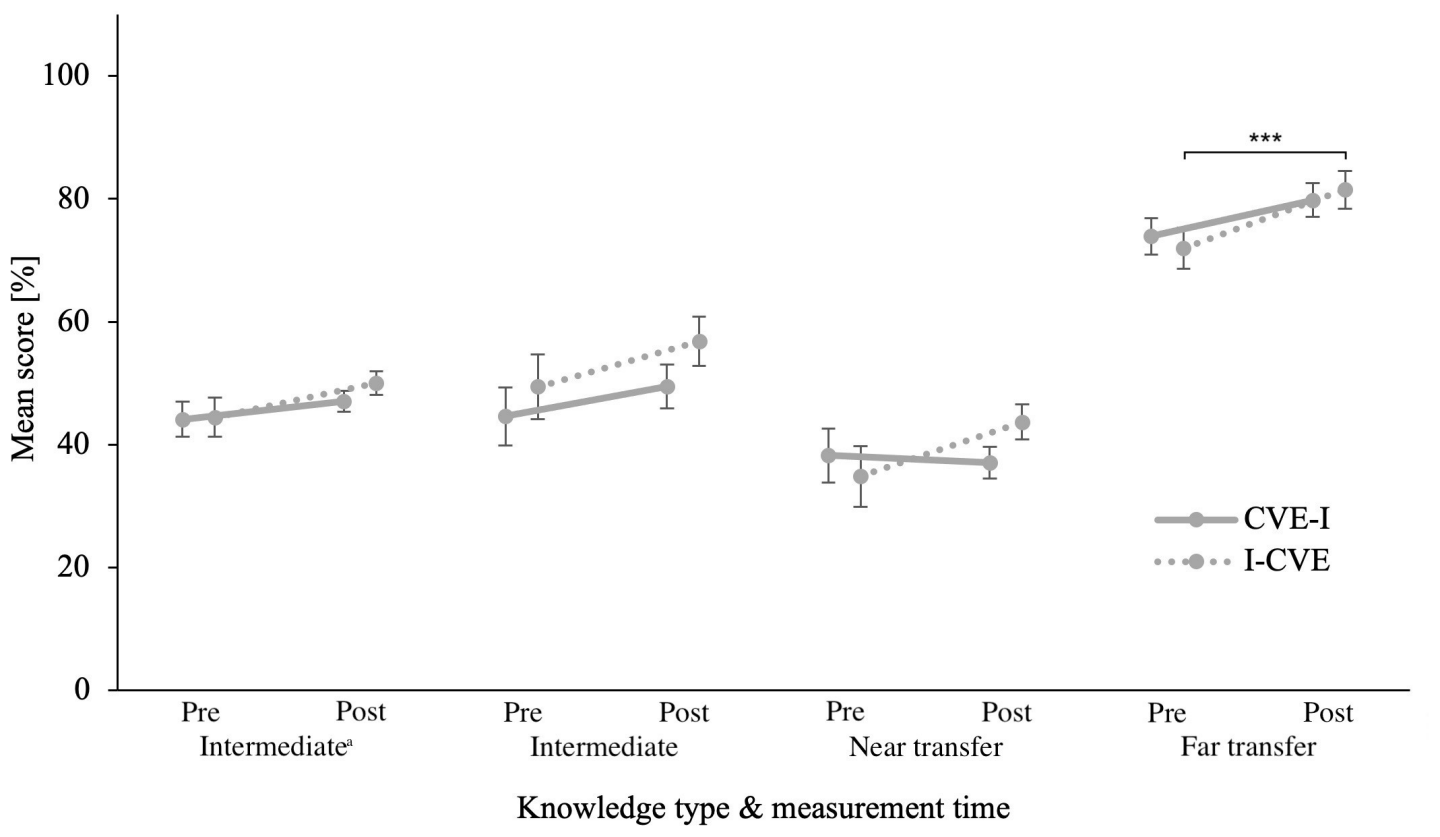
Pre- and Post-intervention Quiz Scores of Conceptual Knowledge in all Modalities. Error bars represent standard error. * p ≤ .05, ** p ≤ .01, *** p ≤ .001 ^a^ Intermediate testing clinical knowledge was assesses after the respective first learning activity during the intervention phase.

The repeated measures ANOVA on *conceptual* CK did not reveal any significant differences, neither for the within-subjects nor for the between-subjects comparison. Corresponding *BF _01_* in this overall trend was 2.00 for pre-post comparison and 3.73 for group comparison. The ANCOVA on post-first learning activity quiz score did not reveal a significant difference between groups, *BF _01_* = 3.06. Please refer to Figure 4 for an illustration for post-first learning activity and post-intervention quiz scores for conceptual knowledge in all quizzes when controlling for the covariates listed in Table 1.

**Figure 5.**
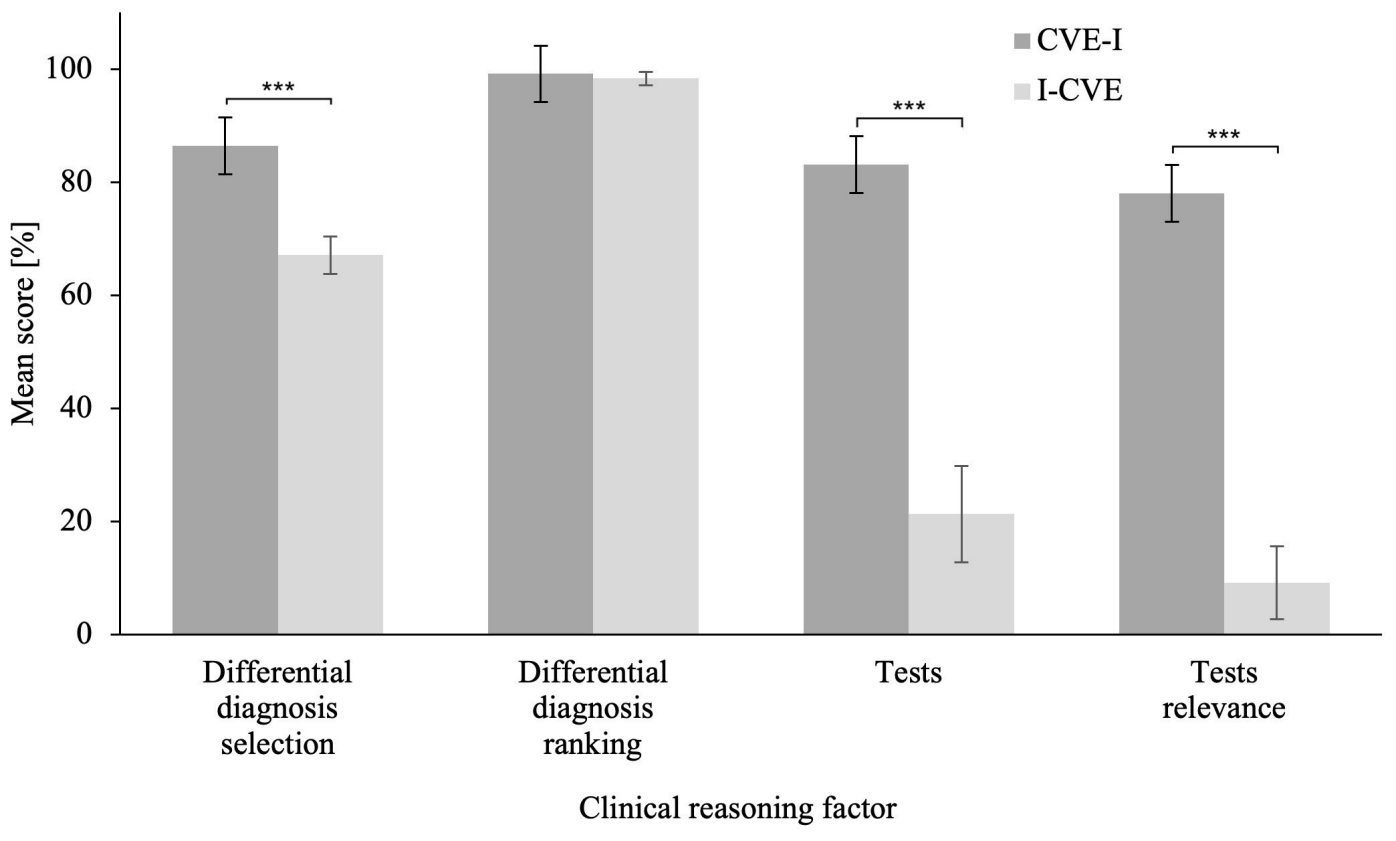
Clinical Reasoning Skills Scores Among Groups in the Intermediate Testing Scenario. The intermediate scenario CRS compares the scores after the respective first learning activity for each group. This was CVE problem-solving for the CVE-I group and direct instruction for the CVE-I group. Error bars represent standard error. * p ≤ .05, ** p ≤ .01, *** p ≤ .001.

The MANCOVA on the intermediate scenario *CRS* revealed a significant difference in CRS scores between groups, *Wilk’s λ* = .442, *F*(4,54) = 17.08, *p* < .001, *η _p_²* = .558. Related univariate ANCOVAs revealed that the CVE-I group significantly outperformed the I-CVE group in Differential Diagnoses Selection, *F*(1,57) = 16.91, *p* < .001, *η _p_²* = .229, *BF _01_* > 100, *ΔM* = 19.33, 95% CI [9.92, 28.74]; Tests, *F*(1,57) = 25.76, *p* < .001, *η _p_²* = .311, *BF _01_* > 100, *ΔM* = 61.79, 95% CI [37.41, 86.17]; and Tests Relevance, *F*(1,57) = 56.90, *p* < .001, *η _p_² =*.500, *BF _01_* > 100, *ΔM* = 68.90, 95% CI [50.61, 87.19]. Please refer to Figure 5 for an illustration of the intermediate testing scenario scores. The ANCOVA on averaged CRS score revealed that the CVE-I group significantly outperformed the I-CVE group, *F*(1,57) = 44.69, *p* < .001, *η _p_²* = .439, *BF _10_* > 100, *ΔM* = 37.71, 95% CI [26.41, 49.00].

### Isomorphic testing

Within-subjects effect analysis of the repeated measures ANOVA on isomorphic *declarative* CK revealed a significant difference between the pre- and post-intervention quiz scores, *F*(1,59) = 16.59, *p* < .001, *η _p_²* = .219. However, between-subjects analysis revealed no significant effects of groups ( Fässler, 2022c). Post-hoc paired samples t-tests using Holm correction, revealed that the I-CVE group performed significantly better in the post-intervention than in the pre-intervention quiz, *ΔM* = 19.44, 95% CI [5.30, 33.59], *t* = 3.75, *p* < .001, but not the CVE-I group. Corresponding *BF _10_* in this overall trend was >100 for pre-post comparison and 0.33 for group comparison. The ANCOVA on post-intervention quiz score did not reveal a significant difference between groups, *BF _01_* = 29.26.

The repeated measures ANOVA on *conceptual* CK did not reveal any significant differences, neither for the within-subjects nor for the between-subjects comparison. Corresponding *BF _01_* in this overall trend was 1.17 for pre-post comparison and 2.03 for group comparison. The ANCOVA on post-intervention quiz score did not reveal a significant difference between groups, *BF _01_* = 59.87.

The MANCOVA on the isomorphic scenario *CRS* scores did not reveal a significant difference between groups. The ANCOVA on averaged CRS confirmed this finding and did not reveal a significant difference between groups neither, *BF _01_ =* 2.94.

### Near transfer testing

Within-subjects effect analysis of the repeated measures ANOVA on near transfer *declarative* CK revealed a significant difference between the pre- and post-intervention quiz scores, *F*(1,59) = 18.49, *p* < .001, *η _p_²* = .239. However, between-subjects analysis revealed no significant effects of groups ( Fässler, 2022b)). Post-hoc paired samples t-tests using Holm correction, revealed that the I-CVE group performed significantly better in the post-intervention than in the pre-intervention quiz, *ΔM* = 21.48, CI [7.67, 35.29], *t* = 4.25, *p* < .001, but not the CVE-I group. Corresponding *BF _10_* in this overall trend was >100 for pre-post comparison and 0.27 for group comparison. The ANCOVA on post-intervention quiz score did not reveal a significant difference between groups, *BF _01_* = 3.82.

The repeated measures ANOVA on *conceptual* CK did not reveal any significant differences, neither for the within-subjects nor for the between-subjects comparison. Corresponding *BF _01_* in this overall trend was 2.77 for pre-post comparison and 4.01 for group comparison. The ANCOVA on post-intervention quiz score did not reveal a significant difference between groups, *BF _01_* = 43.29.

The MANCOVA on the near transfer scenario *CRS* scores did not reveal a significant difference between groups. The ANCOVA on averaged CRS score confirmed this finding and did not reveal a significant difference between groups neither, *BF _10_ =* 0.27.

### Far transfer testing

Within-subjects effect analysis of the repeated measures ANOVA far transfer *declarative* CK did not reveal any significant differences, neither for the within-subjects nor for the between-subjects comparison ( Fässler, 2022a). Corresponding *BF _01_* in this overall trend was 2.78 for pre-post comparison and 2.52 for group comparison. The ANCOVA on post-intervention far transfer quiz score did not reveal a significant difference between groups, *BF _01_* = 3.80.

The repeated measures ANOVA on *conceptual* CK revealed a significant difference between the pre- and post-intervention quiz scores, *F*(1,59) = 13.55, *p* < .001, *η _p_²* = .187. However, between-subjects analysis revealed no significant effects of groups. Post-hoc paired samples t-tests using Holm correction revealed that only the I-CVE group performed better in the post-intervention quiz than in the pre-intervention quiz, *ΔM* = 9.52, CI [0.99, 18.06], *t* = 3.05, *p* = .021, but not the CVE-I group. Corresponding *BF _10_* in this overall trend was 42.31 for pre-post comparison and 0.30 for group comparison. The ANCOVA on post-first learning activity intermediate quiz score did not reveal a significant difference between groups, *BF _01_* > 100.

The MANCOVA on the far transfer scenario *CRS* scores did not reveal a significant difference between groups. The ANCOVA on averaged CRS score confirmed this finding and did not reveal a significant difference between groups neither, *BF _10_ =* 0.28.

### Learning mechanisms

The ANCOVA on feedback response did not reveal a significant difference between groups. The MANCOVA on learning mechanisms did not reveal any significant difference between groups. Univariate ANCOVAs confirmed this finding.

## Discussion

In the present study we aimed to examine the effect of problem-solving in CVEs on CK and CRS isomorphic testing and transfer outcomes and evoked learning mechanisms when combined with direct video instruction in different sequences.

*Problem-solving phase CRS* analysis revealed that I-CVE group significantly outperformed the CVE-I group in all CRS factors. This is a plausible finding because for the I-CVE group the CVE problem-solving was the second learning activity. Hence, the topic was not new for this group. Consequently, this group could work better through the patient scenario by referring to the knowledge, procedures, and concepts taught in the preceding lecture. This resulted in an advantage for the I-CVE group to perform better in this specific scenario. On the other hand, the CVE-I group could not refer to any instruction and were forced to explore the stated problem more to make sense of it.

*Intermediate CK* analysis revealed that both groups significantly improved their declarative CK, but not their conceptual CK. However, there was no significant difference between groups. These findings indicate that some learning has taken place in both learning activities and that they both are effective in imparting declarative knowledge. Furthermore, the absence of a significant difference in the post-first learning activity quiz scores with a *BF _10_* of smaller than 1 indicates that the same content knowledge is taught in both learning activities. The fact that both groups achieved significantly higher scores in CK, especially for declarative CK, after the first learning activity might not be obvious at first view. This is because (declarative) CK was not directly addressed in the problem-solving phase. However, the reason for this finding might be explained by the provided feedback during problem-solving, where some CK and explanations about the patient problem were provided.

*Isomorphic, near transfer, and far transfer CK* and *CRS* analyses revealed no significant difference between the two learning activity sequences in fostering the acquisition of CK and CRS. Even thought, there were no significant differences in neither of the post-intervention CK quizzes, there are indications that the I-CVE learning activity sequence might lead to better acquisition and near transfer of CK, especially for declarative CK. Interestingly, there seems to be hardy any effect on conceptual knowledge.

*Learning mechanisms* analysis did not reveal any significant differences between groups. The lacking difference in triggered learning mechanisms between groups might be explained the fidelity of my CVE-I instructional design to criteria which make the problem-solving prior to instruction approach superior to other instructional designs ( Kapur & Bielaczyc, 2012; Sinha & Kapur, 2021b) of my CVE-I instructional design. First, the CVE problem-solving phase was of heavily scaffolded nature due to the predefined process and the provided instant feedback. Hence, students were guided towards the canonical solution in the CVE phase already. Second, the instruction phase did not build on the solutions the students created in the CVE phase. Third, the instruction phase was represented by a monologue lecture. Based on meta-analytic work by Sinha & Kapur (2021b), these three features are in contrast with common problem-solving prior to instruction designs where – inter alia – (a) lower levels of scaffolding in the problem-solving phase, (b) building on the students’ problem-solving solutions in the instruction phase, and (c) a dialog-dominant nature of the instruction phase have been found to be important aspects which make problem-solving preceding instruction superior to instruction-first approaches. Not meeting these criteria might have resulted in the CVE problem-solving being unable to trigger the assessed learning mechanism better than the instruction. Consequently, because these mechanisms have been posited to be associated with preparatory effects for subsequent instruction, the results of the present study suggest that neither intervention group was prepared better than the other for the respective second learning activity. Finally, this might be the reason why no learning activity sequence led to significantly better post-intervention learning outcomes than the other.

Our findings indicate that only the CVE learning activity imparts CRS and that CRS cannot be taught by theoretical instruction only. Furthermore, despite more failure during preparatory problem-solving, students in the CVE-I condition develop better CRS after the preparatory problem-solving phase compared to students who merely receive instruction on canonical concepts. However, these differences did not translate to post-intervention differences in isomorphic and near and far transfer testing.

Generally, our results should be considered with caution as the standard errors are rather high. Hence, for future research, bigger sample sizes are needed. Furthermore, we did not consider retention of CK and CRS in our study. Consequently, in future studies delayed post-intervention tests should complement the design of the present study.

## Conclusion

For the majority of our post-intervention measures, we did not find significant differences between the CVE-I and I-CVE condition. Consequently, neither of the learning activity sequences fosters the acquisition or near and far transfer of CK and CRS better than the other. When looking at the two learning activities individually, we found that problem-solving in CVE as well as direct instruction are equally effective in imparting content knowledge. However, problem-solving in CVE with formative feedback imparts CRS more effectively than mere instruction. Our study has a high level of ecological validity because it took place in a realistic setting where students had to perform all tasks from home. Distant learning will play an even more important role in the future where students are required to work autonomously.

## Ethical approval

This study was approved by the Ethics Committee of ETH Zürich (reference number 2021-N-24) on March 31, 2021.

## Previous presentations

Short poster presentations were given at the (a) Junior Research (JURE) virtual conference of the European Association for Research on Learning and Instruction (EARLI) in August 2021 and (b) European Association for Medical Education (GMA) virtual conference in September 2021.

## Data Availability

Figshare: FarTransferTesting.xlsx, https://doi.org/10.6084/m9.figshare.21220676.v1 ( Fässler, 2022a). Figshare: NearTransferTesting.xlsx, https://doi.org/10.6084/m9.figshare.21220679.v1 ( Fässler, 2022b). Figshare: IsomorphicTesting.xlsx, https://doi.org/10.6084/m9.figshare.21220688.v1 ( Fässler, 2022c). Figshare: IntermediateTesting.xlsx, https://doi.org/10.6084/m9.figshare.21220685.v1 ( Fässler, 2022d). Figshare: ProblemSolving.xlsx, https://doi.org/10.6084/m9.figshare.21220691.v1 ( Fässler, 2022e). Figshare: ExtendedData1_Figure_ExperimentalDesign.docx, https://doi.org/10.6084/m9.figshare.21221636.v1 ( Fässler, 2022f). Figshare: ExtendedData2_Figure_CVEPlatform.docx, https://doi.org/10.6084/m9.figshare.21221648.v1 ( Fässler, 2022g). Figshare: ExtendedData3_MCQuestions.docx, https://doi.org/10.6084/m9.figshare.21221690.v1 ( Fässler, 2022h). Figshare: ExtendedData4_QuestionnaireItems.docx, https://doi.org/10.6084/m9.figshare.21221738.v1 ( Fässler, 2022i). Figshare: ExtendedData5_Descriptives_All.docx, https://doi.org/10.6084/m9.figshare.21221747.v1 ( Fässler, 2022j). Figshare: ExtendedData6_Descriptives_ANCOVAs.docx, https://doi.org/10.6084/m9.figshare.21221768.v1 ( Fässler, 2022k). Data are available under the terms of the Creative Commons Attribution 4.0 International license (CC-BY 4.0).
