# Problem-solving in virtual environment simulations prior to direct instruction for differential diagnosis in medical education: An experimental study

**DOI:** 10.12688/mep.19348.2

**Published:** 2023-01-23

**Authors:** Christian Fässler, Tanmay Sinha, Christian Marc Schmied, Jörg Goldhahn, Manu Kapur

**Affiliations:** 1 Department of Humanities, Social and Political Sciences, ETH Zürich, Zürich, 8092, Switzerland; 2 Department of Health Sciences and Technology, ETH Zürich, Zürich, 8092, Switzerland; 3 University Heart Center, University Hospital Zürich, Zürich, 8091, Switzerland

**Keywords:** Clinical knowledge, clinical reasoning skills, learning activity sequencing, problem-solving prior to instruction, situated learning, transfer

## Abstract

**Background:** Despite acquiring vast content knowledge about the functioning of the human body through university teaching, medical students struggle to transfer that knowledge to one of the core disciplinary practices – differential diagnosis. The authors aimed to overcome this problem by implementing computer-based virtual environment simulations in medical education courses.

**Methods: **In an experimental study, the authors compared problem-solving in medical computer-based virtual environment simulations prior to instruction with an instruction-first approach. They compared the effects on isomorphic testing and transfer performance of clinical knowledge and clinical reasoning skills as well as evoked learning mechanisms. The study took place in spring 2021 with undergraduate medical students in the scope of a medical trajectory course. Due to Corona-Virus-19 measures participants completed all study activities remotely from home.

**Results:** The authors did not find any learning activity sequence to be superior to the other. However, when looking at the two learning activities individually, they found that problem-solving in computer-based virtual environment simulations and direct instruction might be equally effective for learning content knowledge. Nevertheless, problem-solving in computer-based virtual environment simulations with formative feedback might be more effective for learning clinical reasoning skills than mere instruction.

**Conclusions:** The findings indicate that problem-solving in computer-based virtual environment simulations might be more effective for learning clinical reasoning skills than mere theoretical instruction. The present study has a high level of ecological validity because it took place in a realistic setting where students had to perform all learning and testing tasks autonomously.

## Introduction

Empirical studies reveal that undergraduate medical students struggle on counseling patients in the clinical practice of medicine ( Prince *et al.*, 2005). A major reason for the students’ struggling in practice might be that knowledge which was acquired in a university setting does not transfer to clinical practice ( Collard *et al.*, 2016). Yet, we identify the failure to transfer in current methods of instruction where the focus is on first providing direct instruction on basic knowledge without adequate attention to situate this knowledge in disciplinary practice. First, this is because the majority of cognitive errors are not related to knowledge deficiency, as by the third year of medical school, the average student has acquired an impressive fund of clinical knowledge ( Leeds *et al.*, 2020). Second, there is a significant lack of practical experience during the medical studies, especially in the early years of study ( Diemers *et al.*, 2007; Dornan *et al.*, 2006). Third, if there is work-based training this is often conducted in an isolated way, alongside classroom-based learning, rather than being connected to it ( Peters *et al.*, 2017). Accordingly, to overcome the issue of transfer, we proposed (a) a transition to situations as starting points ( Michaud & Jucker-Kupper, 2017) where learning must be anchored to an actual disciplinary problem and (b) that the acquisition of clinical knowledge and clinical reasoning skills must be situated in disciplinary practice where instruction and practical experience must be closely connected to each other.

According to the new learning objectives catalogue for medical students in Switzerland, situations as staring point are “generic situations which cover the common circumstances, symptoms, complaints and findings that a physician should be able to manage on his first day of residency” ( Michaud & Jucker-Kupper, 2017, p. 26). A specific example of a situation as starting point is headache. However, this situation as starting point might then end in different diagnoses (e.g., migraine, subarachnoid hemorrhage) when going through the differential diagnosis process with a patient. Notably, building on the limitations of past educational methods, the new learning objectives catalogue aims to restructure the medical study curriculum and set situations as starting point as the baseline for learning. Consequently, our study aligns with this approach and our findings might contribute to the current discussion on how the new learning objectives can be implemented into the curricula of medical faculties ( Berendonk *et al.*, 2019) and how they can be assessed objectively.

Whilst we acknowledge that there might be several options for implementing situated learning and situations as starting point and to enhance transfer, we aimed to explore the use of medical computer-based virtual environment (CVE) simulations. Such virtual environment scenarios with simulated patients allow medical students to learn and experience in safe environments from which transfer to other simulated situations or clinical practice can be enhanced. Furthermore, CVE simulations offer a unique possibility to generate almost any relevant medical scenario and align such scenarios with the situations as starting point approach. Additionally, CVE tools and platforms allow individualized learning. Because CVE simulations are scalable and not limited to a certain number of students, their use in medical education is also potentially beneficial for institutions and lecturers from an economic and efficiency perspective in the long-term. However, in empirical research it has yet to be shown how and when CVE simulations are effective in medical education and enhancing transfer.

One method of combining instruction and practical experience to successfully enhance learning and transfer is the so-called *problem-solving prior to instruction (PS-I)* design. This is because recent meta-analytic evidence from the learning sciences suggests that PS-I, on average, results in better conceptual understanding and transfer outcomes than instruction-first learning approaches for comparisons carried out in the domain of medicine ( Sinha & Kapur, 2021b). However, there also are limitations on this approach where the *instruction preceding problem-solving (I-PS)* sequence might be more appropriate. This is why we compared the PS-I with the I-PS learning activity sequence for differential diagnosis education and their effect on clinical knowledge and clinical reasoning skills acquisition and transfer. In our study, problem-solving was represented by a patient scenario in CVE which situates learning in disciplinary practice. In such instructional designs, the first learning activity is assumed to trigger mechanisms which prepare students to benefit from the subsequent learning activity ( Loibl *et al.*, 2017). These learning mechanisms are germane cognitive load, knowledge gap awareness, state curiosity, positive affect and negative affect ( Sinha *et al.*, 2020). Below, we elaborate more on these mechanisms.

In the present study we first focused on *clinical knowledge* which we specify as declarative and conceptual knowledge about specific diseases and the process of differential diagnosis. *Declarative* clinical knowledge describes the recall of specific isolated pieces of knowledge such as facts, definitions, terminologies, concepts, etc. (e.g., Goldman & Hasselbring, 1997) An example related to the present work is a question about the difference between primary and secondary headaches. *Conceptual* knowledge reflects the understanding of interrelationships among facts, procedures, and concepts (e.g., Engelbrecht *et al.*, 2005). An example related to the present work is to understand the meaning of specific measure values in a blood test. Second, we also targeted the underlying mental process of differential diagnosis - clinical reasoning. *Clinical reasoning* describes the thinking and decision-making process associated with clinical practice ( Higgs & Jones, 2008). Clinical reasoning must be applied in every moment of patient attendance and is fundamental for a timely diagnosis of diseases. We annotate that in this study we focused on the strategic aspects to generate differential diagnoses (e.g., using certain procedures and understanding why they might be useful), hence *clinical reasoning skills*, and not on the execution of procedures (e.g., auscultate the heart during the physical examination). For completeness, for the present work clinical reasoning skills are categorized as *procedural* knowledge which reflects the ability to use particular rules related to a concept and to perform a procedure (e.g., Kadijevich & Haapasalo, 2001; Rittle-Johnson *et al.*, 2015).

A broad definition of transfer may be described as applying previously learned knowledge with various degrees of adaptation or modification of that knowledge in completing a task or solving problems. Depending on the degree of the need for adaptation or modification, transfer can be further divided into three levels which include knowledge application, knowledge near transfer, and knowledge far transfer. However, to determine whether a particular testing task assesses near or far transfer is complex because the notion of transfer encompasses several dimensions which are unstated in the definition. Furthermore, this makes it difficult to draw meaningful conclusions from past research because investigators might have use terms of transfer inconsistently ( Barnett & Ceci, 2002). Consequently, we specify our investigations on transfer. Barnett & Ceci (2002) suggest that the characteristics of transfer might be categorized into two overall factors: content and context. The content describes *what* is transferred, whereas the context characterizes *when* and *where* transfer occurs from and to. In our study we investigated the acquisition of clinical knowledge and clinical reasoning skills through two different learning activities in different orders and their subsequent transfer to a similar and dissimilar patient problem. According to the taxonomy of Barnett & Ceci (2002), the content can be – inter alia – divided into two further subdimension: (a) the specificity/generality of the learned skill (procedure, representation, principle or heuristics) and (b) the performance change created by the learning intervention (speed, accuracy, approach). Accordingly, with our study we addressed representation accuracy by assessing clinical knowledge and the approach to (differential diagnosis) principles by assessing clinical reasoning skills. The context can also be broken down into a number of subdimension (c.f. Barnett & Ceci, 2002). However, the only subdimension where there is a remarkable difference between the various testing and transfer problems is the *knowledge domain*. Even though in our study all testing and transfer problems were in the domain of differential diagnosis, this is because these problems were based on different situations as starting point and diagnoses. Consequently, the testing and transfer problems targeted specific knowledge and skills from different disciplines. We acknowledge that our categorization of different kinds of transfer might be oversimplified. However, first due to the fact that among all context subdimensions there is only a remarkable difference between the various testing and transfer problems in *knowledge domai*n and second for better comprehension we define near and far transfer as following. *Near transfer*: means to transfer clinical knowledge and clinical reasoning skills acquired in learning activities based on one situation as starting point, to an assessment method with content building on the *same* situation as starting point but a *different* diagnosis and. *Far transfer:* means to transfer clinical knowledge and clinical reasoning skills acquired in learning activities based on one situation as starting point, to an assessment method with content building on *another* situation as starting points. Furthermore, *isomorphic* means to acquire clinical knowledge and clinical reasoning skills in learning activities based on one situation as starting point and employ it in an assessment method with content building on the *same* situation as starting point and the *same* diagnosis.

In summary, we aimed to evaluate when problem-solving in CVEs in combination with direct instruction can best enhance learning outcomes and how CVEs are effective. We did so by combining problem-solving in CVEs and direct instruction in different orders resulting in the *problem-solving in CVE prior to instruction (CVE-I)* and *instruction prior to problem-solving in CVE (I-CVE)* sequences. We then assessed the effect of the CVE-I and I-CVE sequences on learning mechanisms and the acquisition and transfer of clinical knowledge and clinical reasoning skills. Inter alia, the ability to transfer is important because medical students cannot be confronted with all possible situations in their medical studies they will face later in their professional career as doctors.

## Research questions and hypotheses

To achieve our research goals we stated the four following research questions:

1. How does the sequence of problem-solving in CVE simulations and direct video instruction influence the *learning mechanisms* germane cognitive load, knowledge gap awareness, state curiosity, positive affect, and negative affect?
2. How does the sequence of problem-solving in CVE simulations and direct video instruction facilitate *isomorphic* testing outcomes of clinical knowledge and clinical reasoning skills in *a new disease*?
3. How does the sequence of problem-solving in CVE simulations and direct video instruction facilitate *near transfer* of clinical knowledge and clinical reasoning skills?
4. How does the sequence of problem-solving in CVE simulations and direct video instruction facilitate *far transfer* of clinical knowledge and clinical reasoning skills?

Following, we elaborate more on past research to derive a rational for our hypotheses. As emphasized already, recent meta-analytic evidence suggests that PS-I, on average, results in better conceptual understanding and transfer outcomes than instruction-first learning approaches in the domain of medicine ( Sinha & Kapur, 2021b). However, for procedural knowledge, no such effect could be shown, Hedge’s g = -0.03, 95% CI [-0.20, 0.15] ( Sinha & Kapur, 2021b). For example, Palominos *et al.* (2021) compared the effect of PS-I and I-PS on nursing students learning in healthcare simulations on the acquisition of declarative and explanatory knowledge and transfer of knowledge. They found PS-I to outperform I-PS for explanatory knowledge and the ability of applying learning to solve novel clinical problems but not for declarative knowledge. Furthermore, in their study about sequencing discovery learning and direct instruction for simulation-technical skills, Kulasegaram *et al.* (2018) found that a learning activity sequence resembling PS-I leads to better transfer of simulated suturing skills than I-PS. However, this was not the case for acquisition and retention. Marei *et al.* (2017) combined virtual patient simulations with direct instruction in reversed orders to compare their effect on knowledge acquisition, transfer, and retention and treatment of new virtual patients. They did not find a significant difference between the PS-I (virtual patient simulations preceding instruction) and I-PS sequence regarding their effect on knowledge acquisition and retention. However, for knowledge transfer they found – only for males – the PS-I approach to be more effective for simpler topics, whereas they found the I-PS approach to be more beneficial for more complex topics. Finally, Geel *et al.* (2019) compared a practice before instruction versus an instruction-first approach in X-ray evaluation training. They found the instruction-first (I-PS) sequence to outperform the practice before instruction (PS-I) sequence in diagnostic performance. Remarkably, they indicate that this might be due high relevant prior knowledge of study participants. Consequently, from this study it can be deducted that an I-PS approach could be advised for advanced learners with high prior knowledge. The findings of this might be explained by the expertise reversal effect, a phenomenon where – in simple terms – instructional approaches (such as e.g., PS-I) are highly effective with inexperienced learners but not or even detrimental for more experienced learners ( Kalyuga *et al.*, 2003). These specific examples indicate that beside the beneficial effects of the PS-I sequence on learning outcomes there are also limitations on this sequence where the I-PS sequence might be more appropriate. Especially, this might depend on students’ prior knowledge, the complexity of the tasks or topics, and whether knowledge or skills, or acquisition or transfer shall be fostered.

All studies mentioned above investigated the PS-I versus I-PS sequence on their effect on knowledge and skill acquisition and transfer in the domain of medicine. However, none of these studies has tried to understand why the respective approach might be effective or not. Therefore, we also addressed learning mechanisms which have been posited to be related to the superior learning outcomes in PS-I compared to an instruction-first approach ( Glogger-Frey *et al.*, 2015; Lamnina & Chase, 2019; Loibl *et al.*, 2017). Specifically, we assessed knowledge gap awareness, state curiosity, germane cognitive load, and positive and negative affect. Regarding *knowledge gap awareness*, existing review of PS-I literature suggests that students need to be made aware of the limitations to their knowledge ( Loibl *et al.*, 2017). This is because it has been postulated that students who better perceive their knowledge gaps might be better prepared to fill these knowledge gaps by receiving instruction ( Loibl *et al.*, 2017). Importantly, it is indicated that PS-I leads to higher knowledge gap awareness than I-PS designs ( Loibl & Rummel, 2014). Additionally, *state curiosity* is an important motivational factor in problem-solving because strong state curiosity is related to the desire to know more and to fill knowledge gaps ( Glogger-Frey *et al.*, 2015). Related thereto, Lamnina & Chase (2019) indicate that uncertainty provokes state-curiosity. In our study, this uncertainty might be increased through problem-solving in CVE rather than through direct instruction. *Germane cognitive load* results from instructional formats that increase cognitive load as well as learning by relating relevant information from long-term memory to new information elements ( Leppink *et al.*, 2014; Sweller, 1988). Activities that promote high germane load might facilitate learning and contribute to transfer performance by helping to build correct mental models ( Paas *et al.*, 2003). For example, such an activity might be to solve problems in an applied task such as a simulated patient encounter. Therefore, high germane load indicates that learners are engaged and direct their mental resources to learning processes. Sinha & Kapur (2021a) indicated that PS-I can be expected to evoke *positive affect* (i.e., surprise, interest, and confusion). Furthermore, Lamnina & Chase (2019) indicated that there is a positive relationship between self-reported positive affect and learning outcomes, especially for isomorphic problem-solving testing. On the other hand, negative affect (i.e., anger, disgust, and contempt) might also have beneficial effect on learning outcomes in PS-I designs ( Sinha & Kapur, 2021a). This is because “experiencing moderate levels of negative emotions keeps one alerted of challenges requiring more focused attention, and assists in comprehending conflicting information” ( Sinha, 2022, p. 41). Such negative affect can be evoked by designing an ill-structured problem-solving nature ( Sinha, 2022) where in our study this might be reflected by a patient problem in CVE.

Related to research question 1, the studies mentioned above indicate that CVE-I sequence in general might be more beneficial for transfer but not for acquisition of procedural knowledge and skills. Furthermore, the learning mechanisms have been shown to beneficial for conceptual knowledge and transfer. Consequently, learning mechanisms might explain why these two learning outcomes are fostered in the CVE-I sequence. However, there are also studies including virtual patient simulations in PS-I designs which did not find any learning activity sequence to be superior to the other on knowledge acquisition and transfer. Accordingly, the effectiveness of a PS-I or I-PS instructional approach might rather dependent on prior knowledge and the complexity of the task topic. Consequently, the better triggering of learning mechanisms in the CVE-I sequence might be impeded by factors such as task complexity or prior knowledge. Consequently, we hypothesized a similar triggering of learning mechanisms after the first learning activity for both groups.

*Learning mechanisms*

1.1 There will not be any difference between learning activity sequences in *germane cognitive load*.
1.2 There will not be any difference between learning activity sequences in *knowledge gap awareness*.
1.3 There will not be any difference between learning activity sequences in *state curiosity*.
1.4 There will not be any difference between learning activity sequences in *positive affect*.
1.5 There will not be any difference between learning activity sequences in *negative affect*.

As specified, the learning mechanisms addressed above are assumed to trigger cognitive processes which prepare students to benefit from subsequent instruction. Based on our hypotheses 1.1 – 1.5 that these mechanisms will be triggered to a similar extent among experimental groups, we deducted that students of both groups must be prepared for the respective for the second learning activity to a similar degree. Furthermore, studies have shown that a PS-I instructional approach in general might be more beneficial for transfer but not for acquisition of procedural knowledge and skills. For our study, this would indicate that CVE-I might be superior to I-CVE for conceptual clinical knowledge acquisition and clinical reasoning skills transfer. Additionally, research indicates that the PS-I sequence (CVE-I) might be more beneficial for students with low prior knowledge. Accordingly, as the intervention and isomorphic testing focused on a novel disease for students, their prior knowledge might be assumed to be low. Consequently, for our study this further supports – particularly for third-year medical students with low prior knowledge on a certain topic – that the CVE-I sequence might lead to better conceptual clinical knowledge acquisition and better clinical knowledge and clinical reasoning skills transfer than the I-CVE sequence. Thus, we made the following hypotheses on learning outcomes related to research questions 2 to 4.

*Isomorphic testing*

2.1 CVE-I will lead to similar or slightly better isomorphic testing outcomes of *clinical knowledge* in a new disease than I-CVE.
2.2 CVE-I will lead to similar or slightly better isomorphic testing outcomes of *clinical reasoning skills* in a new disease than I-CVE

*Near transfer testing*

3.1 CVE-I will lead to similar or slightly better near transfer of *clinical knowledge* than I-CVE.
3.2 CVE-I will lead to similar or slightly better near transfer of *clinical reasoning skills* than I-CVE.

*Far transfer testing*

4.1 There will not be any difference between learning activity sequences in far transfer of *clinical knowledge*.
4.2 CVE-I will lead to similar or slightly better far transfer of *clinical reasoning skills* than I-CVE.

## Methods

### Participants

The present study took place in scope of a third-year medical course at an open admission highly ranked university in Western Europe. Students were randomly assigned to the intervention groups before the course start. We could recruit *N* = 61 (63.93% female, *n* = 39) students who gave written (online) informed consent to include their data in the study whereof *n* = 34 (58.82% female, *n* = 20) students were assigned to the CVE-I group and *n* = 27 (70.37% female, *n* = 19) to the I-CVE group. No compensation was given to study participants. This study was approved by the Ethics Committee of ETH Zurich.

### Study context

Due to Coronavirus-19 ( World Health Organization (WHO), 2020) measures, the study took place online. The students completed all activities of the study autonomously from home on I-Human Patients, Moodle, and Qualtrics. However, we sent detailed instructions to students via e-mail about the proceeding of the next study phase or day.

### Experimental design

**Figure 1.**
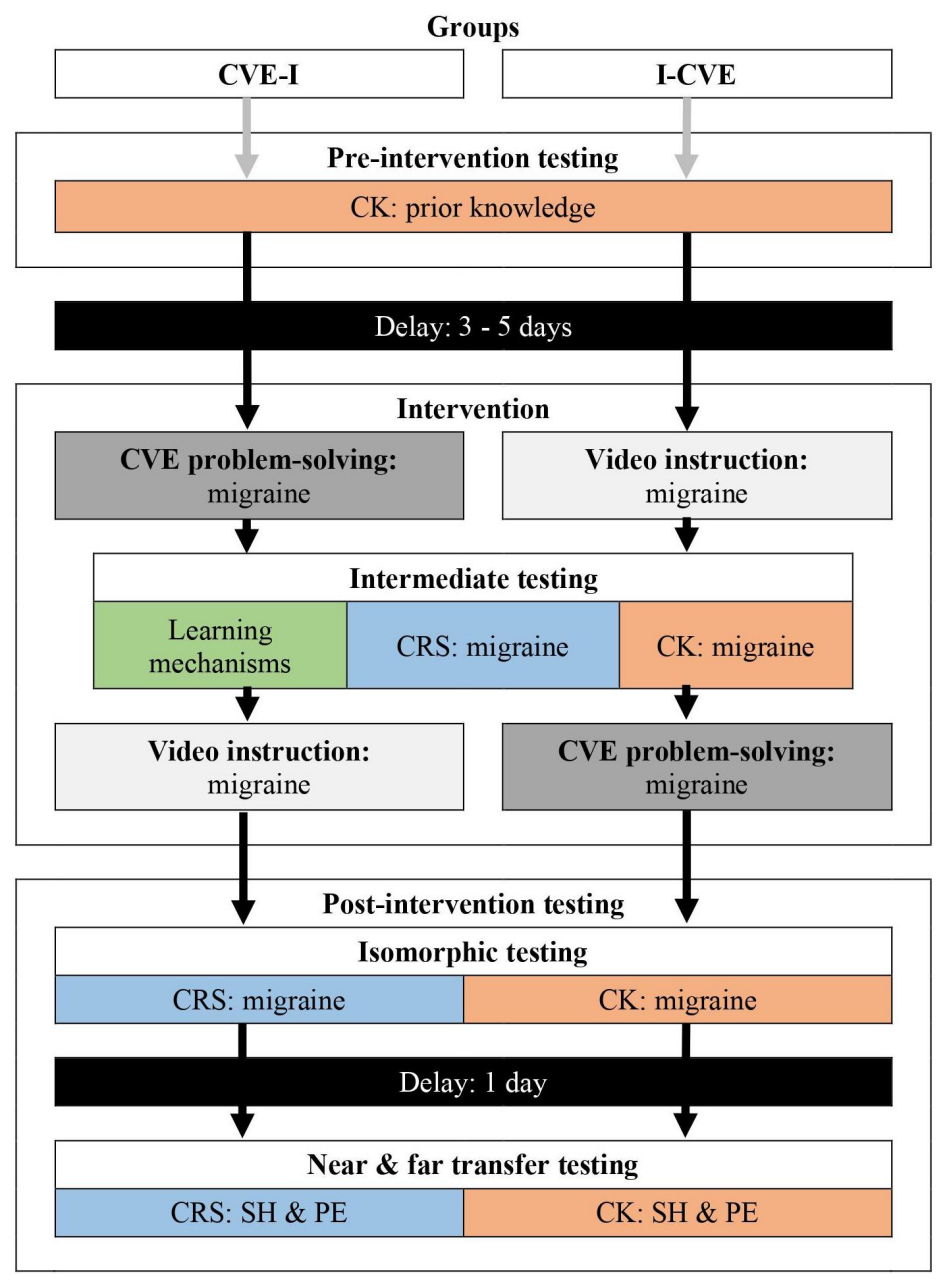
Experimental Design. Green illustrates assessments of learning mechanisms, red of clinical knowledge, and blue of clinical reasoning skills. Abbreviations: CK: clinical knowledge; CRS: clinical reasoning skills; CVE: computer-based virtual environment; PE: pulmonary embolism; I: direct video instruction (video lecture); SH: subarachnoid hemorrhage.

There were two subject groups going either through the *CVE-I* or *I-CVE* learning activity sequence in the intervention phase. The pre-intervention phase took place three to five days prior to the intervention. The intervention phase took place within one morning where participants followed their learning activities and intermediate testing according to their assigned sequence. The post-intervention phase took place over two days due to time considerations. In the afternoon of the intervention day, participants went through the isomorphic assessment. On the next day, students first went through the near transfer assessment and then through the far transfer assessment. Please refer to Figure 1 for an illustration of the experimental design.

### Learning materials and measures

**Figure 2.**
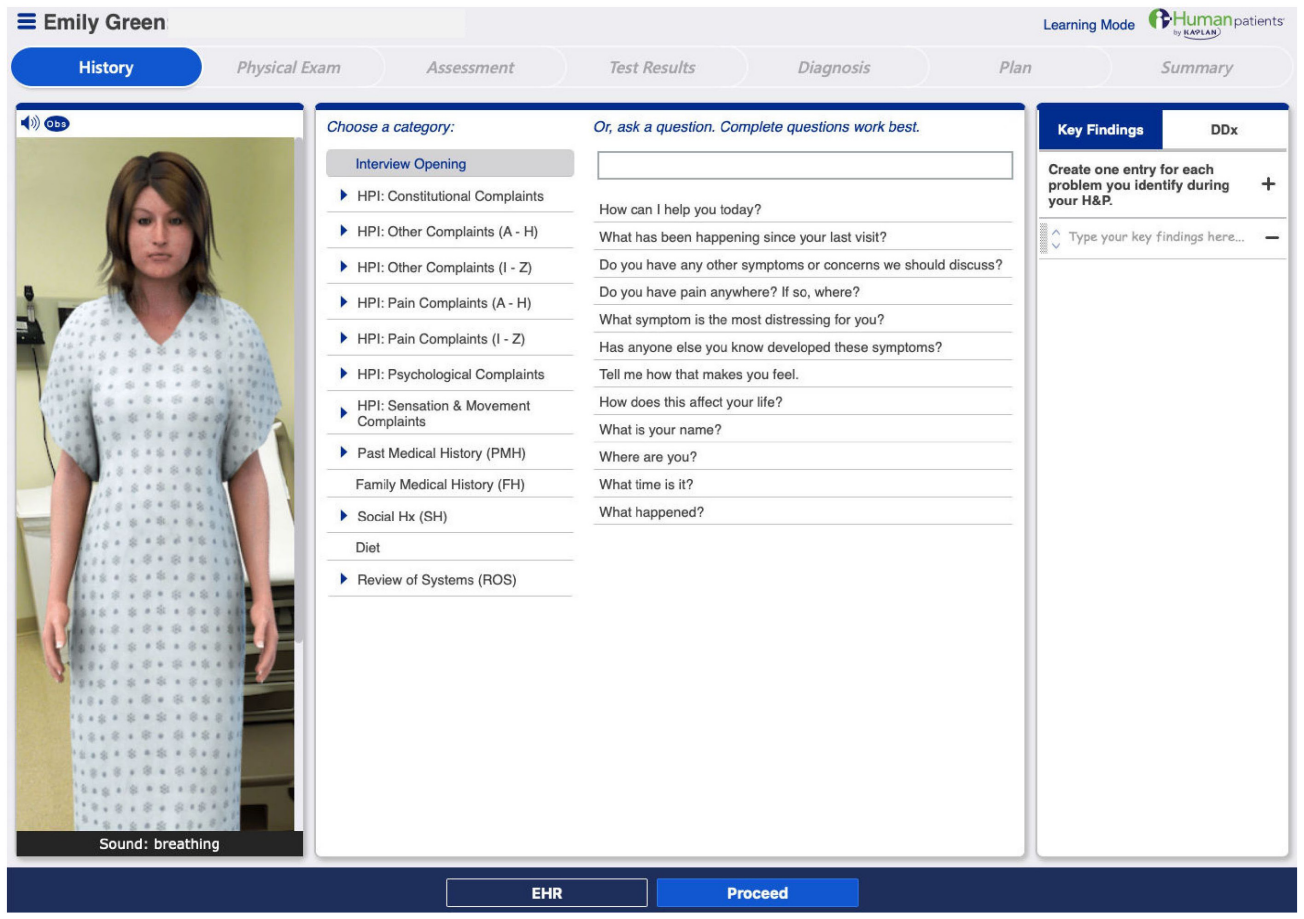
Implemented CVE Platform I-Human Patients. Figure reprinted with permission of I-Human Patients by Kaplan, https://www.i-human.com

***CVE platform***. We used the interactive learning platform I-Human Patients for our medical CVE simulations. Please refer to Figure 2 for an illustration of the platform. To assess and quantify clinical reasoning skills we extracted the following measures from the CVE scenarios:

- *History*: asking correct questions when obtaining the patient’s history
- *History Relevance*: correctly asked questions in relation to extraneous and missed ones
- *Physical Examination*: selecting correct physical examinations during the patient encounter based on findings in the patient history
- *Physical Examination Relevance*: correctly selected examination in relation to extraneous and missed ones
- *Differential Diagnoses Selection*: selecting correct eligible differential diagnoses based on findings in the patient history and physical examination (or electronic patient record)
- *Tests*: selecting correct clinical tests to include/exclude eligible differential diagnoses
- *Tests Relevance*: correctly selected clinical tests in relation to extraneous and missed clinical ones
- *Time*: time used to solve the CVE scenario

***Pre-intervention phase***. In this phase, students had to solve a multiple-choice quiz with questions related to the medical topics covered in the intervention and post-intervention phase. This pre-intervention quiz was to assess students’ prior knowledge and to establish the baseline for potential clinical knowledge acquisition and transfer caused by the intervention. Consequently, all questions of the pre-intervention quiz were asked again later in the study process when assessing the corresponding modality. We took quiz questions from the I-Human Patients platform because they are properly matched to the content of the CVE scenarios. Multiple-choice questions were set up and answered in Moodle. We assessed clinical knowledge in four modalities with multiple-choice questions: (a) intermediate, (b) isomorphic, (c) near transfer, and (d) far transfer. Please refer to the sections «Intermediate testing» and «Post-intervention testing» for question examples. Furthermore, please refer to Extended Data 1 ( Fässler, 2022f) for all implemented questions. Scores of all multiple-choice quizzes were transformed into percentage values.

***Problem-solving phase***. In this phase, students worked independently through the CVE problem-solving scenario. The simulated differential diagnosis process consisted of four stages: (a) taking the patient history by asking questions to the patient, (b) performing the physical examination by selecting appropriate examinations based on the findings in the history part, (c) selecting eligible differential diagnoses based on findings in the patient history and physical examination, and (d) selecting appropriate clinical tests to include/exclude eligible differential diagnoses. In the problem-solving scenario, individualized instant formative feedback was provided after each of these four stages. The problem-solving scenario was based on a simulated patient with the situations as starting point headache where the differential diagnosis process ended with migraine.

***Instruction phase***. This phase was represented by a monologue video lecture of 25 minutes duration. This lecture consisted of (a) a theoretical introduction where a doctor provided information about headaches and the corresponding diagnostic approach and (b) a case review where the doctor worked through the differential diagnosis process on an actual patient with migraine. The video lecture was provided via the I-Human Patients platform.

***Intermediate testing***. After the first learning activity, we collected students’ self-reported learning mechanisms measurements on a five-point Likert scale via questionnaires in Qualtrics: *germane cognitive load* ( Leppink *et al.*, 2014; 6 items, e.g., “This activity improved my understanding of the content that was covered *”*), *knowledge gap awareness* ( Glogger-Frey *et al.*, 2017; 5 items, e.g., “My knowledge was insufficient to carry out these tasks”), *state curiosity* ( Naylor, 1981; 9 items, e.g., “I feel like asking questions about what is happening”), *positive affect* ( Watson *et al.*, 1988; 10 items, e.g., determined, enthusiastic) and *negative affect* ( Watson *et al.*, 1988; 10 items, e.g., upset, distressed). Please refer to Extended Data 2 ( Fässler, 2022g) for all questionnaire items. We also introduced (a) an *intermediate* multiple-choice quiz on Moodle (9 items, e.g., “What is the difference between a primary and secondary headache?”) and (b) a CVE testing scenario on I-Human Patients which was shorter than the standard scenarios. Like the problem-solving scenario, the intermediate testing scenario was based on the situations as starting point headache leading to migraine. However, the patient looked differently and the patient history and physical exam were replaced by an electronic patient record from which students had to extract the most relevant information to continue with the differential diagnosis process. No feedback was provided.

***Post-intervention testing***. This phase was split into three sections: isomorphic, near transfer and far transfer testing. Each of these three sections consisted of a CVE scenario and a multiple-choice quiz. During the isomorphic testing section, participants worked through the same scenario as in problem-solving scenario. However, the patient looked different and no feedback was provided. This was followed by an isomorphic multiple-choice quiz with questions about migraine (10 questions, e.g., “Your patient describes her/his headache as preceded by an aurea, diplopia, and loss of coordination. What is the most likely diagnosis?”). During the near transfer testing section, participants worked through a patient scenario which was also based on the situations as starting point headache but led to subarachnoid hemorrhage. This was followed by a near transfer multiple-choice quiz with questions about subarachnoid hemorrhage (4 questions, e.g., “You suspect that your patient has a subarachnoid hemorrhage, but the non-contrast CT is negative. Which test would be best to perform next?”). During the far transfer testing section, participants worked through a patient scenario which was based on the situations as starting point chest pain and lead to pulmonary embolism. This was followed by a far transfer multiple-choice quiz with questions about pulmonary embolism (6 questions, e.g., “Which of the following organ systems should be considered when investigating the cause of dyspnea?”).

### Analysis plan

For all statistical analyses we used the IBM SPSS, version 27 (IBM Corporation, Armonk, New York), and JASP, version 0.15 (Department of Psychological Methods, University of Amsterdam, Amsterdam), computer software. Before carrying out the analyses, we checked whether randomization of students to groups was effective. We did so by applying a Mann-Whitney U test on pre-intervention quiz scores for each clinical knowledge modality. As alternative complementary analyses for null hypothesis significance testing, we used Bayesian informative hypotheses evaluation and corresponding non-parametric approaches in all analyses ( Hoijtink *et al.*, 2019). This allowed direct evaluation of the Bayes factor of a null hypothesis ( *BF _01_*) at hand or equivalence testing versus its alternative hypothesis ( *BF _10_*) for measure differences. Some students did not work through all study activities. Hence, datasets for these students were incomplete. Therefore, we ran multiple imputations (n = 5) analysis on all included variables. Subsequently, we ran Mann-Whitney U tests on each variable with an incomplete dataset to compare observed and pooled means where we were looking for significant differences between these two values.

***Learning mechanisms***. We conducted an ANCOVA, controlling for average pre-intervention clinical knowledge, to compare feedback response in the problem-solving scenario. Furthermore, we conducted a MANCOVA, boot-strapped with 1000 replications, to compare the extent to which the posited learning mechanisms were triggered among the CVE-I and I-CVE groups after the respective first learning activity.

***Clinical knowledge***. We ran individual ANCOVAs on intermediate (after the first learning activity) and post-intervention isomorphic, near transfer, and far transfer quiz scores, controlling for pre-intervention clinical knowledge in the respective modality, to compare the performance in clinical knowledge testing after the intervention between groups. Notably, these analyses were performed for declarative and conceptual knowledge questions separately. This was because the intervention might influence these two kinds of knowledge differently.

***Clinical reasoning skills***. First, we ran a MANCOVA, boot-strapped with 1000 replications, on problem-solving scenario clinical reasoning skills scores, controlling for Time used to solve this scenario and overall pre-intervention clinical knowledge. Second, we ran individual MANCOVAs, boot-strapped with 1000 replications, on the intermediate, isomorphic, near transfer and far transfer scenario, controlling for Time used to solve the respective scenario and overall pre-intervention clinical knowledge in the respective modality. This was to compare the performance in each CVE scenario between groups. To get a complete overview, we averaged the scores of all clinical reasoning skills factors in each scenario (problem-solving, intermediate, isomorphic, near transfer, and far transfer). We then ran ANCOVAs, boot-strapped with 1000 replications, on the averaged score of each scenario, controlling for Time in the respective scenario and overall pre-intervention quiz score in the respective modality. The ANCOVA on the problem-solving scenario controlled for Time used to solve this scenario and overall pre-intervention clinical knowledge.

## Results

Multiple imputations analysis revealed that there were three missing values for all near transfer clinical reasoning skills factors, one missing value for all far transfer clinical reasoning skills factors, two missing values for the reaction to provided feedback during the problem-solving scenario, and two missing values for all learning mechanisms. Comparison of observed and pooled means did not reveal a significant difference between these two values for any of the imputed variables, *BF _10_* was ≤ 0.20 for all comparisons. Please refer to Extended Data 3 for descriptives of the analyses ( Fässler, 2022h). Hence, we did not exclude any student from the analyses. Consequently, we ran the analyses with all students and the corresponding available data. Nevertheless, the number of students ( *N*) included varied among analyses. Please note that figures with data representations in red illustrate information about clinical knowledge and in blue about clinical reasoning skills.

### Pre-intervention testing

The Mann-Whitney U test on average pre-intervention quiz score did not reveal a significant difference between groups. Bayesian Mann-Whitney U testing confirmed this finding, *BF _01_* = 2.80. When considering prior clinical knowledge in all modalities individually, no significant difference between groups was revealed in neither of the clinical knowledge modalities. Bayesian Mann-Whitney U testing confirmed this finding, intermediate clinical knowledge, *BF _10_* = 0.36; isomorphic clinical knowledge, *BF _10_* = 0.29; near transfer clinical knowledge, *BF _10_* = 0.48; far transfer clinical knowledge, *BF _10_* = 0.58. Consequently, this is anecdotal to moderate evidence that groups were equal and randomization was effective. Please refer to Extended Data 4 ( Fässler, 2022i) for descriptives of prior knowledge and all other measures included in the present study.

### Problem-solving phase

**Figure 3.**
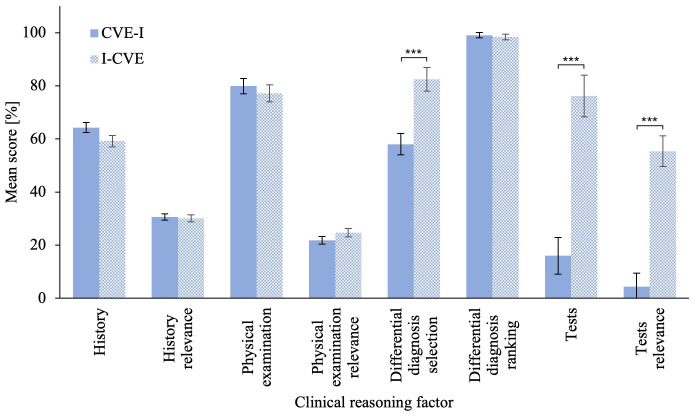
Clinical Reasoning Skills Scores in the Problem-Solving Scenario. The problem-solving phase clinical reasoning skills compares the scores of the first learning activity for the CVE-I group and of the second learning activity for the I-CVE group. Error bars represent standard error. * p ≤ .05, ** p ≤ .01, *** p ≤ .001.

**Figure 4.**
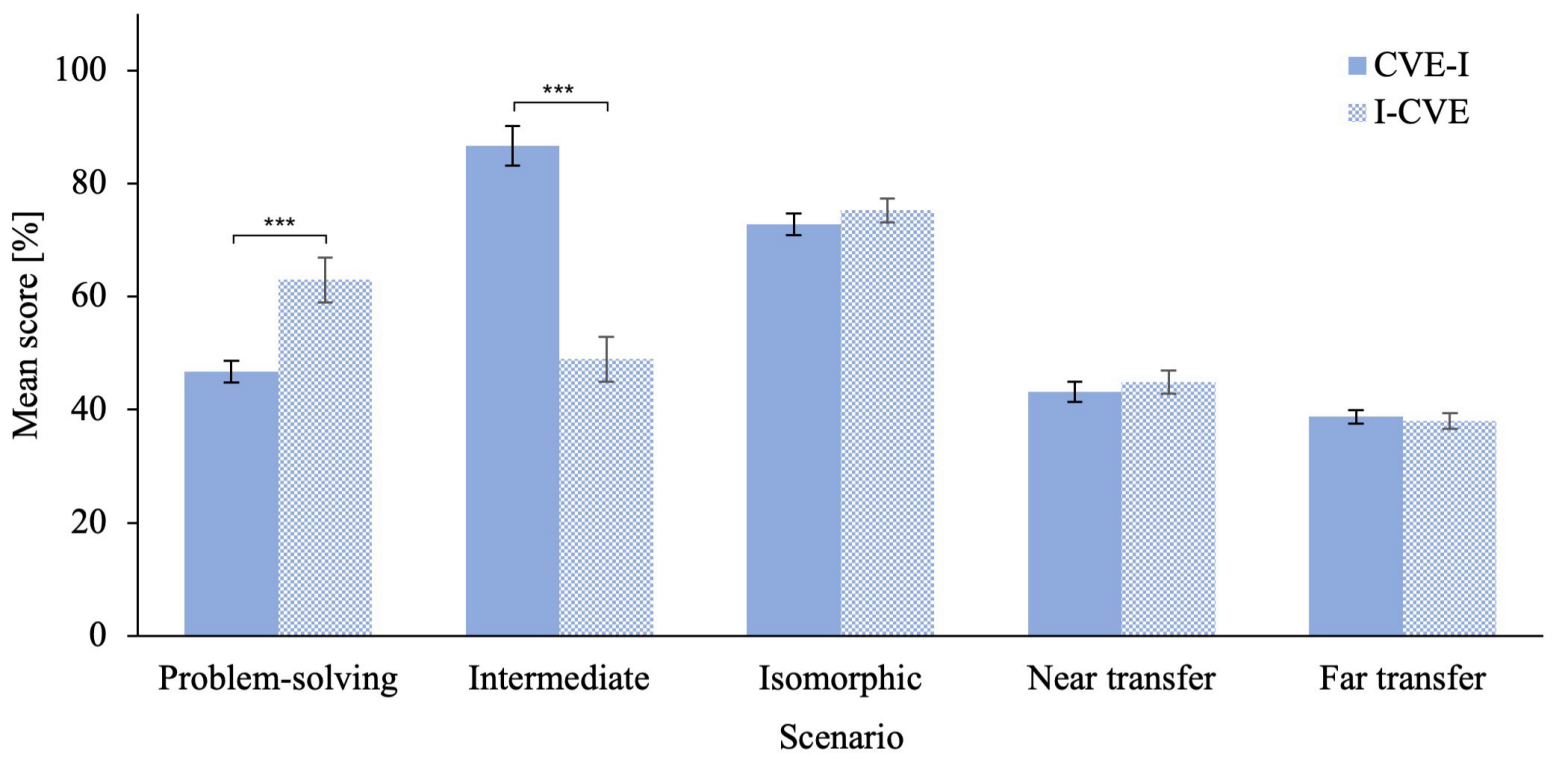
Averaged Score of Clinical Reasoning Skills Factors for Each Scenario. Error bars represent standard error. * p ≤ .05, ** p ≤ .01, *** p ≤ .001.

The MANCOVA on the problem-solving scenario revealed a significant difference in clinical reasoning skills score between groups, *Wilk’s λ* = .482, *F*(8,50) = 6.73, *p* < .001, *η _p_²* = .518, observed power > .99 ( Fässler, 2022e). Related univariate ANCOVAs revealed that the I-CVE group significantly outperformed the CVE-I group in Differential Diagnoses Selection, *F*(1,57) = 15.99, *p* < .001, *η _p_²* = .219, *BF _10_* = 56.78, observed power = .98, Δ *M* = 24.46, 95% CI [12.21, 36.70]; Tests, *F*(1,57) = 31.51, *p* < .001, *η _p_²* = .356, *BF _10_* > 100, observed power > .99, Δ *M* = 60.14, 95% CI [38.69, 81.59]; and, Tests Relevance, *F*(1,57) = 41.47, *p* < .001, *η _p_²* = .421, *BF _10_* > 100, observed power > .99, Δ *M* = 51.03, 95% CI [35.16, 66.90]. Please refer to Figure 3 for an illustration of the problem-solving scenario score. The ANCOVA on averaged clinical reasoning skills score revealed a that the I-CVE group significantly outperformed the CVE-I group, F(1,57) = 29.11, p < .001, *η _p_²* = .338 *, BF _10_* > 100, observed power > .99, ΔM = 16.18, CI [10.18, 22.19]. Pre-intervention quiz score was moderately related to average clinical reasoning skills score, *F*(1,57) = 3.61, *p* = .062, *η _p_²* = .060, *BF _10_* = 1.44, observed power = .46. Time was not significantly related to average clinical reasoning skills score, *BF _10_* = 0.37. Please refer to Figure 4 for an illustration of the ANCOVA results for all CVE scenarios. Furthermore, please refer to Extended Data 5 ( Fässler, 2022j) for descriptives of the ANCOVAs on all CVE scenarios.

### Intermediate testing

**Figure 5.**
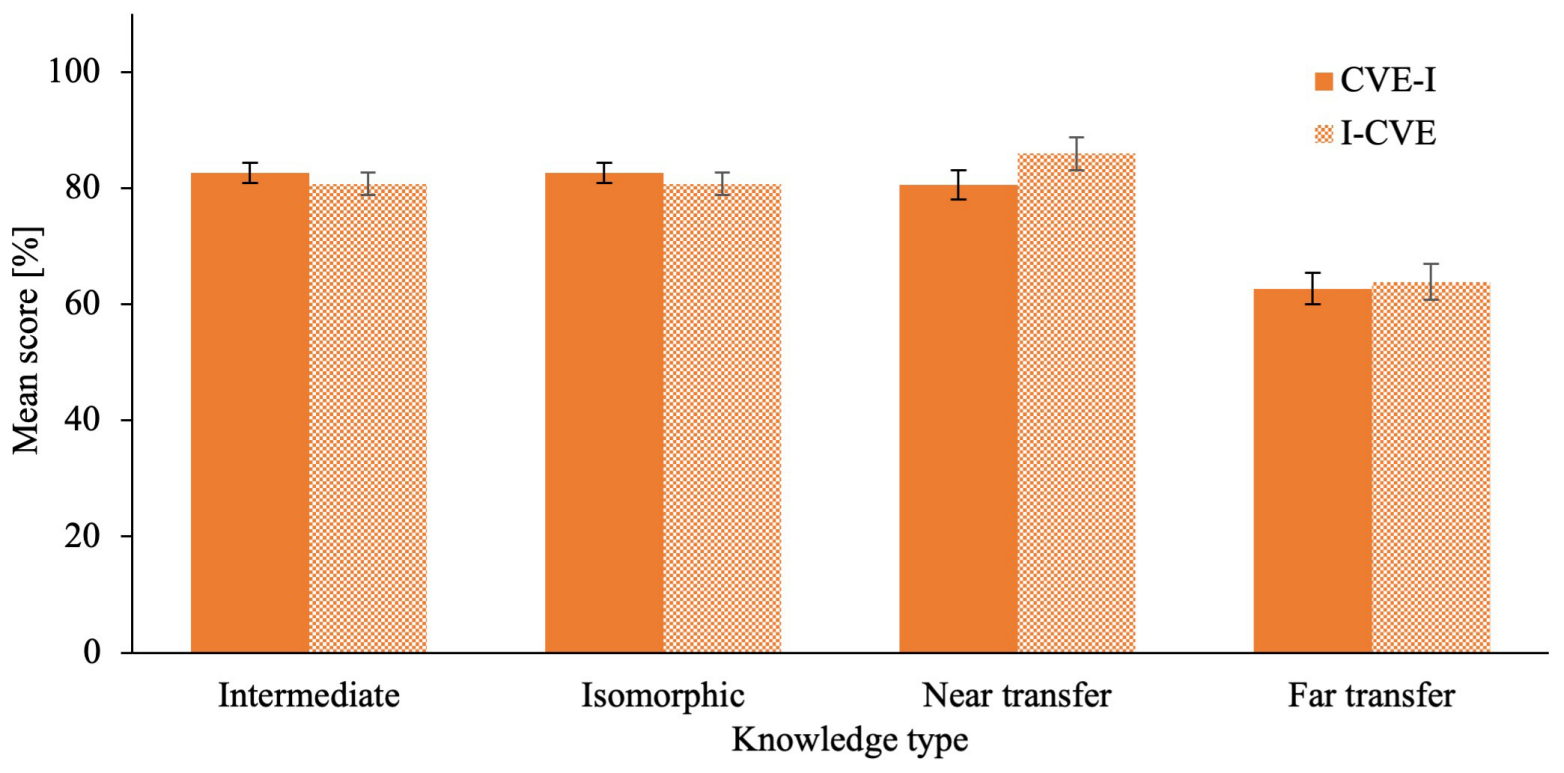
Post- intervention Quiz Scores of Declarative Knowledge in all Modalities. Error bars represent standard error. * p ≤ .05, ** p ≤ .01, *** p ≤ .001 ^a^ Intermediate testing clinical knowledge was assesses after the respective first learning activity during the intervention phase.

**Figure 6.**
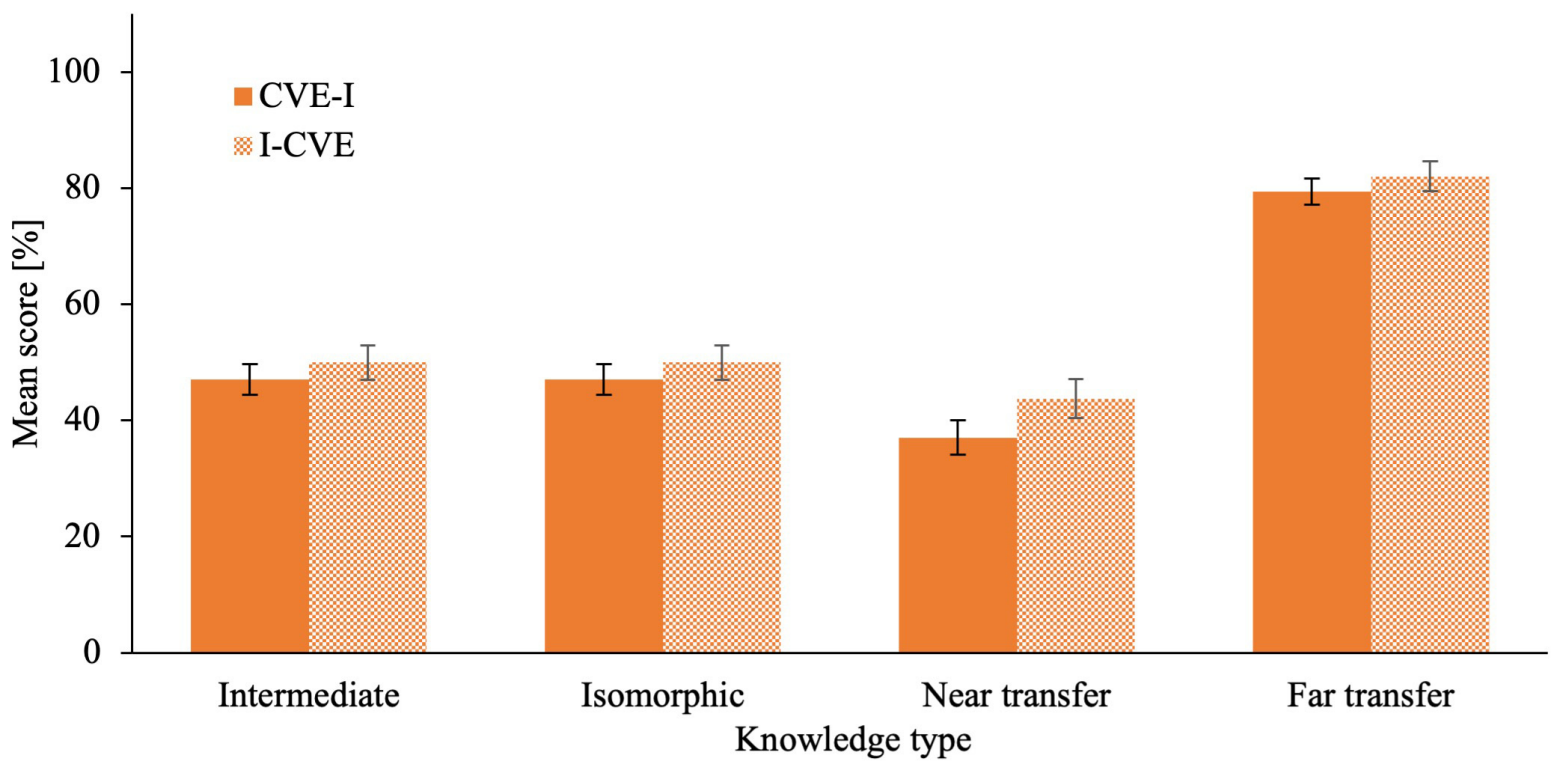
Post-intervention Quiz Scores of Conceptual Knowledge in all Modalities. Error bars represent standard error. * p ≤ .05, ** p ≤ .01, *** p ≤ .001 ^a^ Intermediate testing clinical knowledge was assesses after the respective first learning activity during the intervention phase.

The ANCOVA on intermediate *declarative* clinical knowledge quiz score did not reveal a significant difference between groups, *BF _10_* = 0.36 ( Fässler, 2022d). Pre-intervention quiz score was not significantly correlated to post-intervention quiz score, *BF _10_* = 0.67. Cronbachs’α for the declarative clinical knowledge quiz was .532, 95% CI [.278, .726]. Please refer to Figure 5 for an illustration for post-first learning activity and post-intervention quiz scores for declarative knowledge in all quizzes. The ANCOVA on intermediate *conceptual* clinical knowledge quiz score did not reveal a significant difference between groups, *BF _10_* = 0.36. Pre-intervention quiz score was not significantly related to post-intervention quiz score, *BF _10_* = 0.67. Cronbachs’α for the conceptual clinical knowledge quiz was .508, 95% CI [.100, .990]. Please refer to Figure 6 for an illustration for post-first learning activity and post-intervention quiz scores for conceptual knowledge in all quizzes.

**Figure 7.**
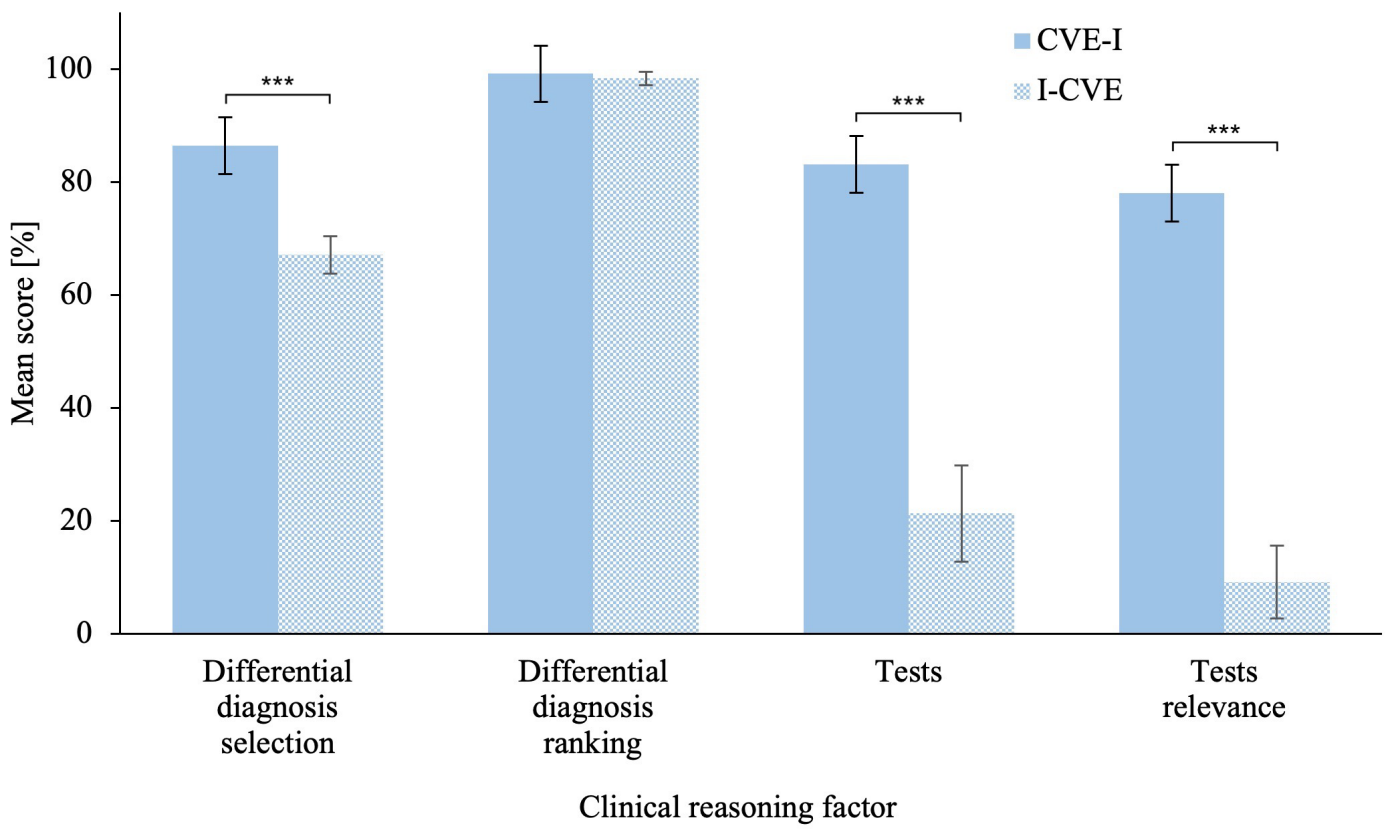
Clinical Reasoning Skills Scores Among Groups in the Intermediate Testing Scenario. The intermediate scenario CRS compares the scores after the respective first learning activity for each group. This was CVE problem-solving for the CVE-I group and direct instruction for the CVE-I group. Error bars represent standard error. * p ≤ .05, ** p ≤ .01, *** p ≤ .001.

The MANCOVA on the intermediate scenario *clinical reasoning skills* revealed a significant difference in clinical reasoning skills score between groups, *Wilk’s λ* = .442, *F*(4,54) = 17.08, *p* < .001, *η _p_²* = .558, observed power > .99. Related univariate ANCOVAs revealed that the CVE-I group significantly outperformed the I-CVE group in in Differential Diagnoses Selection, *F*(1,57) = 16.91, *p* < .001, *η _p_²* = .229, *BF _10_* > 100, observed power = .98, *ΔM* = 19.33, 95% CI [9.92, 28.74]; Tests, *F*(1,57) = 25.76, *p* < .001, *η _p_²* = .311, *BF _10_* > 100, observed power > .99 *ΔM* = 61.79, 95% CI [37.41, 86.17]; and Tests Relevance, *F*(1,57) = 56.90, *p* < .001, *η _p_² =*.500, *BF _10_* > 100, observed power > .99, *ΔM* = 68.90, 95% CI [50.61, 87.19]. Please refer to Figure 7 for an illustration of the intermediate testing scenario scores. The ANCOVA on averaged clinical reasoning skills score revealed that the CVE-I group significantly outperformed the I-CVE group, *F*(1,57) = 44.69, *p* < .001, *η _p_²* = .439, *BF _10_* > 100, *ΔM* = 37.71, 95% CI [26.41, 49.00], observed power > .99. Neither pre-intervention quiz score, *BF _10_* = 0.63, nor Time, *BF _10_* = 0.41.was significantly related to average clinical reasoning skills score.

### Learning mechanisms (research question 1)

The ANCOVA on feedback response did not reveal a significant difference between groups, *BF _10_* = 0.28. The MANCOVA on learning mechanisms did not reveal any significant difference between groups. Univariate ANCOVAs confirmed this finding.

### Isomorphic testing (research question 2)

The ANCOVA on isomorphic *declarative* clinical knowledge quiz score revealed a moderately significant difference between groups, *F*(1,58) = 3.70, *p* = .059, *η _p_²* = .060, *BF _10_* = 1.16, observed power = .92 ( Fässler, 2022c). Pre-intervention quiz score was significantly related to post-intervention quiz score *F*(1,58) = 11.16, *p* = .001, *η _p_²* = .161, *BF _10_* = 19.36, observed power = .91. Cronbachs’α for the declarative clinical knowledge quiz was .403, 95% CI [.094, .633]. The ANCOVA isomorphic *conceptual* clinical knowledge quiz score did not reveal a significant difference between groups, *BF _10_* = 0.17. Cronbachs’α for the conceptual clinical knowledge quiz was .394, 95% CI [.004, .643].

The MANCOVA on the isomorphic scenario *clinical reasoning skills* scores did not reveal a significant difference between groups. The ANCOVA on averaged *clinical reasoning skills* confirmed this finding and did not reveal a significant difference between groups neither, *BF _10_ =* 0.34.

### Near transfer testing (research question 3)

The ANCOVA on near transfer *declarative* clinical knowledge quiz score did not reveal a significant difference between groups, *BF _10_* = 0.48 ( Fässler, 2022b). Cronbachs’α for the declarative clinical knowledge quiz was .541, 95% CI [.255, .075]. The ANCOVA on near transfer *conceptual* clinical knowledge quiz score did not reveal a significant difference between groups, *BF _10_* = 0.68. Cronbachs’α for the conceptual clinical knowledge quiz was .427, 95% CI [.022, .649].

The MANCOVA on the near transfer scenario *clinical reasoning skills* scores did not reveal a significant difference between groups. The ANCOVA on averaged clinical reasoning skills score confirmed this finding and did not reveal a significant difference between groups neither, *BF _10_ =* 0.29.

### Far transfer testing (research question 4)

The ANCOVA on far transfer *declarative* clinical knowledge quiz score did not reveal a significant difference between groups, *BF _10_* = 0.27 ( Fässler, 2022a). Cronbachs’α for the declarative clinical knowledge quiz was .405, 95% CI [.060, .662]. The ANCOVA on far transfer *conceptual* clinical knowledge quiz score did not reveal a significant difference between groups, *BF _01_* = 0.32. Cronbachs’α for the conceptual clinical knowledge quiz was .330, 95% CI [.002, .761].

The MANCOVA on the far transfer scenario *clinical reasoning skills* scores did not reveal a significant difference between groups. The ANCOVA on averaged clinical reasoning skills score confirmed this finding and did not reveal a significant difference between groups neither, *BF _10_ =* 0.28.

## Discussion

In the present study we aimed to examine the effect of problem-solving in CVEs on (a) clinical knowledge and clinical reasoning skills isomorphic testing and transfer outcomes (b) and evoked learning mechanisms when combined with direct video instruction in different sequences.

*Problem-solving phase clinical reasoning skills* analysis revealed that I-CVE group significantly outperformed the CVE-I group in all clinical reasoning skills factors. This is a plausible finding because for the I-CVE group the CVE problem-solving was the second learning activity. Hence, the topic was not new for this group. Consequently, this group could work better through the patient scenario by referring to the knowledge, procedures, and concepts taught in the preceding lecture. This resulted in an advantage for the I-CVE group to perform better in this specific scenario. On the other hand, the CVE-I group could not refer to any instruction and were forced to explore the stated problem more to make sense of it.

*Intermediate clinical knowledge* analysis revealed no significant difference between groups, neither for declarative nor for conceptual clinical knowledge. Hence, both learning activities individually might be equally effective for learning clinical knowledge. Furthermore, the absence of a significant difference in the post-first learning activity quiz scores with a *BF _10_* of smaller than 1 indicates that the same content knowledge is taught in both learning activities. Referring to the taxonomy of Barnett & Ceci (2002) this also suggests that both learning activities might improve representation accuracy of clinical knowledge.

*Intermediate* clinical reasoning skills analysis revealed that the CVE-I group outperformed the I-CVE group in three out of four clinical reasoning factors. This is a reasonable finding because the intermediate testing took place after the respective first learning activity and the I-CVE group could only relate to theoretical knowledge because they went through direct instruction first. However, the CVE-I group could refer to experiences and feedback in the problem-solving scenario. Even though the same content knowledge was taught in both learning activities (see above) the CVE-I group performed significantly better than the I-CVE group. This finding indicates that problem-solving in CVE simulations might be more effective for learning clinical reasoning skills than theoretical instruction. Referring to the taxonomy of Barnett & Ceci (2002) this also suggests that problem-solving in CVE simulations might improve the approach to differential diagnosis principles better than mere instruction.

*Isomorphic, near transfer, and far transfer clinical knowledge* and *clinical reasoning skills* analyses revealed no significant difference between the two learning activity sequences. These findings support our hypotheses 2.1 – 4.2. Referring to the taxonomy of Barnett & Ceci (2002) these findings also suggests that neither learning activity sequences is statistically superior to the other for improving representation accuracy of clinical knowledge and approach to differential diagnosis principles.

At this stage we would also like to refer on aspects of multimedia learning which can be described as a form of computer-aided instruction that uses visual and audio modalities concurrently. This is because students were heavily engaged in multimedia presentations through their problem-solving activity in the CVE simulations. Particularly, we focus on one of the principles of multimedia learning established by Mayer (2009) and its application in computer games and simulations for instruction ( Mayer, 2021). Mayer’s principles suggest how to design multimedia materials to effectively enhance learning. Notably, these suggestions heavily rely on aspects of the cognitive load theory ( Sweller, 1988), aiming to minimize extraneous and optimize intrinsic and germane cognitive load. Considering learning activity sequencing, the pre-training principle might be particularly important to explain the lacking difference between the experimental groups in the post-intervention testings. The pre-training principle indicates that students learn more sustainably from multimedia materials when they are familiar with the main concepts ( Mayer, 2009). This is because the complexity of information in a new multimedia message might overwhelm novice students due to cognitive overload ( Mayer, 2009). Particularly, such an overload might occur in fast-paced narrated animations in which students have to construct a mental causal model of the explained concept and the concept’s key components. Related thereto, Mayer & Fiorella (2021) indicate that a pre-training (such as a short instruction) can help the processing of essential information in a simulation or game by distributing the cognitive demands. Notably, the pre-training is stated be associated with prior knowledge activation ( Mayer & Fiorella, 2021). Consequently, both approaches, multimedia learning and PS-I, are effective due to some preparatory activity to learn from a subsequent activity where this preparation might be caused through the activation of prior knowledge (for PS-I, c.f. Loibl *et al.*, 2017) and modulation of cognitive load. However, the difference is that research on multimedia learning claims some sort of instruction to be the preparatory activity for learning from subsequent educational games and simulations ( Mayer & Fiorella, 2021). On the other hand, research on PS-I claims some problem-solving (which in our study was game-/simulation-based) to be the preparatory activity for subsequent instruction ( Sinha & Kapur, 2021b). Both approaches have their merits as the effectiveness of both have been shown in several studies. However, in relation to our study the positive effects of both approaches might have cancelled each other out which finally ended up in no learning activity sequence to be superior to the other. This is because CVE-I can be rather associated with the PS-I approach, whereas I-CVE rather corresponds to the concept of multimedia learning. Please refer to section “Limitations and future work” for further elaboration on the missing differences in post-intervention testings.

Our findings indicate that problem-solving in CVE simulations might be more effective for learning clinical reasoning skills than mere theoretical instruction. Furthermore, despite more failure during preparatory problem-solving, students in the CVE-I condition develop better clinical reasoning skills after the preparatory problem-solving phase compared to students who merely receive instruction on canonical concepts. However, these differences did not translate to post-intervention differences in isomorphic and near and far transfer testing.

### Limitations and future work

First, learning mechanisms analysis did not reveal any significant differences between groups. These findings support our hypotheses 1.1 – 1.5. Because these mechanisms have been posited to be associated with preparatory effects for subsequent instruction ( Sinha *et al.*, 2020), results of the present study suggest that neither intervention group was prepared better than the other for the respective second learning activity. Finally, this might be one cause that none of the two intervention groups was able to outperform the other. An obvious option to modify learning mechanisms induction would be to adapt the learning activities. First, this might be implemented through non-canonical problem-solving ( Loibl & Rummel, 2014) as for example less facilitation, guidance, or feedback provision. Second, direct instruction might include contrasting examples to the canonical solution ( Loibl & Rummel, 2014).

Second, our findings might be explained by three principles which were indicated to make PS-I superior to I-PS ( Sinha & Kapur, 2021b). Notably, these principles were not met in the CVE-I condition. Even though not meeting these criteria was not detrimental for learning it might have prevented the CVE-I (PS-I) group from outperforming the I-CVE (I-PS) group. First, the CVE problem-solving phase was of heavily scaffolded nature due to the predefined process and the provided instant feedback. Hence, students were guided to the canonical solution in the CVE problem-solving phase already. Second, the instruction phase did not build on the solution attempts the students created in the CVE phase. Third, the instruction phase was represented by a monologue lecture. Based on meta-analytic work by ( Sinha & Kapur, 2021b), these three principles as applied in our study are in contrast with common effective PS-I designs where (a) lower levels of scaffolding in the problem-solving phase, (b) building on the students’ problem-solving solutions in the instruction phase, and (c) a dialog-dominant nature of the instruction phase have been found to be important aspects which make PS-I superior to I-PS. Consequently, one possibility to lower the level of scaffolding is to omit the provision of feedback or change its timing.

Third, we would like to conceive the work of Kapur (2011) about facilitation during problem-solving prior to instruction. In his study, Kapur (2011) compared complex problem-solving without instructional facilitation to such problem-solving with facilitation and a traditional lecture-and-practice approach. Notably, CVE-I as applied in our design might represent facilitated complex problem-solving as there is provided feedback during the CVE problem-solving phase. On the other hand, I-CVE might represent the traditional approach where an instruction is followed by facilitated problem-solving. Kapur (2011) found no significant differences between facilitated complex problem-solving prior to instruction and the traditional lecture and practice approach on learning outcomes such as well-structured and higher-order application problems. Notably, this is what we found in our study too. There was no significant difference between the CVE-I and I-CVE group in any of the assessed learning outcomes after the intervention. Nevertheless, Kapur (2011) and Loibl & Rummel (2014) both emphasized that in PS-I, guidance during the initial problem-solving phase might lead to better performance directly after problem-solving compared to no guidance. However, this better initial superiority finally does not prepare students to learn better from a subsequent instruction. Aligning this suggestion with the findings of our study (that there was no significant difference between groups in post-intervention testing) might indicate that even though CVE-I students were failing in the problem-solving phase (this is because their scores in the problem-solving scenario were much lower than in the isomorphic testing scenario and lower than those in the problem-solving scenario of the I-CVE students), they were not prepared well enough to learn sufficiently from the subsequent instruction to finally outperform the I-CVE group. Hence, neither group was prepared better than the other to learn from the respective second learning activity. Consequently, from this accordance to the work of Kapur (2011) and Loibl & Rummel (2014). We deduct that the facilitation during initial problem-solving might a considerable factor that CVE-I was not superior to I-CVE. Consequently, reducing facilitation in the problem-solving phase might lead to better learning outcomes.

Fourth, outside of the PS-I approach, several variables have been shown to influence the effectiveness of formative feedback on learning (e.g., Shute, 2008). Particularly, the research of Ambrose *et al.* (2010) on feedback in simulations indicated that the ideal timing of feedback cannot be determined by any general rule. However, research addressing the timing of formative feedback during problem-solving specifically in the PS-I design is limited. Nevertheless, research focuses on scaffolding and guidance in the problem-solving phase prior to instruction in general. For example, in their recent meta-analysis about preparatory approaches for future learning, Sinha & Kapur (2021c) compared standard problem-solving prior to instruction approaches with such approaches that specifically consisted scaffolded problem-solving phases. They indicated that scaffolded problem-solving, including strategy/outcome feedback, can be regarded as a more substantial preparation for subsequent instruction because of deliberate student involvement in solution creation and revision. Connecting feedback to scaffolding, feedback is touted as one form of guidance that may be particularly effective. Based on their meta-analysis, Alfieri *et al.* (2011) recommended specifically timed feedback provision as an optimal form of guidance. Although the research above is not directly related to timing of feedback in a problem-solving prior to instruction approach and to the field of medical education and differential diagnosis, the literature above suggests that different timings of feedback during problem-solving might lead to different outcomes on learning, such as knowledge and skills acquisition and transfer. Consequently, future research might specifically address the timing of provided formative feedback during the problem-solving phase in the PS-I instructional design and investigate the corresponding effect on learning mechanisms and specific learning outcomes.

Finally, our results should be considered with caution as the standard errors are rather high. Hence, for future research, bigger sample sizes are needed. Furthermore, we did not consider retention of clinical knowledge and clinical reasoning skills in our study. Investigating long term effects the two learning activity sequences might be important because long-term effects of learning outcomes might be different from short-term effects in PS-I instructional designs (e.g., Ziegler *et al.*, 2021) Consequently, in future studies delayed post-intervention tests should complement the design of the present study.

## Conclusion

For the majority of our post-intervention measures, we did not find significant differences between the CVE-I and I-CVE group. Consequently, neither of the learning activity sequences fosters the acquisition or near and far transfer of clinical knowledge and clinical reasoning skills better than the other. When looking at the two learning activities individually, we found that problem-solving in CVE as well as direct instruction are equally effective for learning content knowledge. However, problem-solving in CVE with formative feedback might be more effective for learning clinical reasoning skills than mere instruction. Our study has a high level of ecological validity because it took place in a realistic setting where students had to perform all tasks from home. Distant learning will play an even more important role in the future where students are required to work autonomously.

## Ethical approval

This study was approved by the Ethics Committee of ETH Zürich (reference number 2021-N-24) on March 31, 2021.

## Previous presentations

Short poster presentations were given at the (a) Junior Research (JURE) virtual conference of the European Association for Research on Learning and Instruction (EARLI) in August 2021 and (b) European Association for Medical Education (GMA) virtual conference in September 2021.

## Data Availability

Figshare: FarTransferTesting.xlsx, https://doi.org/10.6084/m9.figshare.21220676.v1 ( Fässler, 2022a). Figshare: NearTransferTesting.xlsx, https://doi.org/10.6084/m9.figshare.21220679.v1 ( Fässler, 2022b). Figshare: IsomorphicTesting.xlsx, https://doi.org/10.6084/m9.figshare.21220688.v1 ( Fässler, 2022c). Figshare: IntermediateTesting.xlsx, https://doi.org/10.6084/m9.figshare.21220685.v1 ( Fässler, 2022d). Figshare: ProblemSolving.xlsx, https://doi.org/10.6084/m9.figshare.21220691.v1 ( Fässler, 2022e). Figshare: ExtendedData1_MCQuestions.docx, https://doi.org/10.6084/m9.figshare.21221690.v2 ( Fässler, 2022f). Figshare: ExtendedData2_QuestionnaireItems.docx, https://doi.org/10.6084/m9.figshare.21221738.v2 ( Fässler, 2022g). Figshare: ExtendedData3_Descriptives_MI.docx, https://doi.org/10.6084/m9.figshare.21221648.v3 ( Fässler, 2022h). Figshare: ExtendedData4_Descriptives_All.docx, https://doi.org/10.6084/m9.figshare.21221747.v3 ( Fässler, 2022i). Figshare: ExtendedData5_Descriptives_ANCOVAs.docx, https://doi.org/10.6084/m9.figshare.21221768.v3 ( Fässler, 2022j). Data are available under the terms of the Creative Commons Attribution 4.0 International license (CC-BY 4.0).
