# Supplementary material for: Problem-solving in virtual environment simulations prior to direct instruction for differential diagnosis in medical education: An experimental study
Source: MedEdPublish (2016). 2023 Jan 23;12:61. Originally published 2022 Oct 19. [Version 2] doi: 10.12688/mep.19348.2 (PMC9925172; doi:10.12688/mep.19348.2)
Supplement: Peer review history

# Peer review: Reviewer response for version 2

**DOI:** 10.21956/mep.20857.r32913

**Published:** 2023-01-25

**Authors:** Lennart Schalk

**Affiliations:** 1 Institute for Research on Instruction and Subject-Specific Education, PH Schwyz, Goldau, Switzerland

The authors did a gread job in revising their research article based on the reviewers' queries and suggestions. The minor issues that I had with the first submission are resolved entirely. I do not have any further issues which would require another revision.

Have any limitations of the research been acknowledged?

Partly

Is the work clearly and accurately presented and does it cite the current literature?

Yes

If applicable, is the statistical analysis and its interpretation appropriate?

Partly

Are all the source data underlying the results available to ensure full reproducibility?

Yes

Is the study design appropriate and is the work technically sound?

Yes

Are the conclusions drawn adequately supported by the results?

Partly

Are sufficient details of methods and analysis provided to allow replication by others?

Yes

Reviewer Expertise:

Learning and instruction; cognitive psychology; STEM education

I confirm that I have read this submission and believe that I have an appropriate level of expertise to confirm that it is of an acceptable scientific standard.

# Peer review: Reviewer response for version 2

**DOI:** 10.21956/mep.20857.r32912

**Published:** 2023-01-24

**Authors:** Christian Hartmann

**Affiliations:** 1 Department of Educational Sciences, Technical University of Munich, Munich, Germany

The authors have fully addressed my comments/aspects from the last round of review. I have no further comments.

Have any limitations of the research been acknowledged?

Partly

Is the work clearly and accurately presented and does it cite the current literature?

Yes

If applicable, is the statistical analysis and its interpretation appropriate?

Partly

Are all the source data underlying the results available to ensure full reproducibility?

Yes

Is the study design appropriate and is the work technically sound?

Yes

Are the conclusions drawn adequately supported by the results?

Yes

Are sufficient details of methods and analysis provided to allow replication by others?

Yes

Reviewer Expertise:

Virtual Reality, Multimedia Learning, Computer-assisted Learning, Example-based Learning, Computer-supported Collaborative Learning, Productive Failure

I confirm that I have read this submission and believe that I have an appropriate level of expertise to confirm that it is of an acceptable scientific standard.

# Peer review: Reviewer response for version 1

**DOI:** 10.21956/mep.20733.r32674

**Published:** 2022-11-15

**Authors:** Christian Hartmann

**Affiliations:** 1 Department of Educational Sciences, Technical University of Munich, Munich, Germany

The manuscript titled " *Problem-solving in virtual environment simulations prior to direct instruction for differential diagnosis in medical education: An experimental study*“ presents an interesting and important study related to research on problem-solving prior to instruction approaches (hereafter referred to as PS-I). Even though the current version of the manuscript is of good quality and I recommend it passing peer review, I have some major and minor comments on the current version of the manuscript. I will list these in the order in which I perceived them while reading.

1. The manuscript works with a lot of abbreviations (e.g., CK, CVE, CRS, DD, SSP, CK). In my opinion, these disturb the reading flow and unnecessarily complicate understanding. Therefore, I would like to encourage the authors to write out the abbreviations completely and use them only in tables, if necessary at all.
2. In the introduction, on page 3, the authors state: “Y *et, we identify the failure to transfer in current methods of instruction where the focus is on first providing direct instruction on basic knowledge without adequate attention to situate this knowledge in disciplinary practice.*” Even if an introduction does not have to provide all arguments in their full extent, I would have expected a bit more elaborated discussion here. For instance, why are PS-I approaches better than instruction-first approaches for the educational setting the authors are addressing? I have the impression that it would be sufficient to emphasize the main arguments more clearly when presenting the research objectives throughout the introduction.
3. Concerning the theory used, I have the further remark that so far no references to the theory of multimedia learning have been made (e.g., “multimedia principles” outlined by Meyer). In the present study, PS-I was investigated in a virtual learning environment that relies on simulations. Accordingly, research on PS-I needs also to be discussed with respect to learning settings, in which learners engage in multimedia presentations such as simulations. Even though I am aware that it is not necessary to discuss the present setting entirely against the background of multimedia learning (the research question is rather directed towards PS-I),  reflecting the virtual setting used against the background of PS-I research would strenghten the theoretical presentation. For instance, I wonder whether assumed PS-I mechanisms work differently in a virtual multimedia setting as used in the present study. Are there studies with comparable (virtual) learning settings? If not in the theoretical introduction, such a section could conceivably be included in the discussion section.
4. On page 4, the authors mention that the virtual learning environment provided feedback to the learners: “In the problem-solving scenario, individualized instant formative feedback was provided after each of these four stages.” I would like to encourage the authors to specify and also discuss a bit more on how this might have affected results or PS-I mechanisms. To the best of my knowledge, providing feedback during the problem-solving phase is not foreseen in PS-I and as PS-I results seem to be the main theoretical/empirical framework here, this aspect needs more attention. This might be best placed in a (to be added) limitation section. If the authors decide to integrate a limitation section (what I highly recommend), there are further limitations which might be interesting to discuss, for instance the online format of the study or the domain of the learning materials as well as the targeted knowledge. Also, I would like to see a discussion whether PS-I effects can be compared to the learning setting presented here. For instance, a discussion of PS-I fidelity criteria based on the authors previous work might be a suitable approach addressing this. This can be either done in the discussion or limitation section.
5. I would have expected a report of estimated statistical power for the main hypotheses tests, for instance in the analysis plan. I would like to ask the authors to provide these analyses (either post-hoc or - if previously performed - a priori).
6. May add a figure/illustration of the experimental procedure. Even if the procedure is nicely described, this might help readers to get an easier access to the essential phases of the experiment. Also, pictures/screenshots of the used virtual learning platform might nicely illustrate the learning scenario.
7. Parameters to determine the reliability of the measurements (e.g., internal consistency) were not provided. I would like to ask the authors to add Cronbach's alpha (or comparable parameters) for all scales used.
8. The hypotheses might be better introduced by presenting them explicitly. In the current state, the hypotheses are rather passively presented. This might be done in an own section prior to the analysis plan or alternatively as a part of the analyses plan, that is, where specific hypotheses tests were discussed in detail.
9. In the method section, the authors state: “However, we did not exclude any student from the analysis because multiple imputation did not have a significant influence on the results.” Even if I (intuitively) agree with the authors conclusion, I would have expected a bit more information on the imputation method and why the authors argue that the imputation did not have a significant influence. This aspect would be sufficiently addressed by some further information on the authors perspective on this issue.
10. Finally, I would like to note that I agree with the other reviewer's comment on the analyses used, i.e., the suggestion to rely on ANCOVAs for the analyses. I would like to add one point to this. I wonder why so many covariates were used? Are the conditions for considering covariates violated or appropriate for all of them, e.g., do all covariates correlate with the outcome (equally in both conditions) or are the means of the covariates for both conditions equivalent? I am aware that this is a controversial methodological discussion, and based on the current presentation, I find the authors' analyses sound. However, I would encourage the authors to provide a little more information regarding the requirements of the use of covariates (either through a response to my comment or in a footnote to the table in the manuscript). In the event that the conditions for integrating individual covariates are violated (which I do not anticipate) and the authors find this problematic, they should consider removing affected covariates from the analyses, as this could potentially decrease, rather than increase, the interpretability of the ANCOVA results. For a discussion, see: Culpepper, S. A., & Aguinis, H. (2011). Using analysis of covariance (ANCOVA) with fallible covariates. Psychological Methods, 16(2), 166-178. https://doi.org/10.1037/a0023355 ^1^.

Have any limitations of the research been acknowledged?

Partly

Is the work clearly and accurately presented and does it cite the current literature?

Yes

If applicable, is the statistical analysis and its interpretation appropriate?

Partly

Are all the source data underlying the results available to ensure full reproducibility?

Yes

Is the study design appropriate and is the work technically sound?

Yes

Are the conclusions drawn adequately supported by the results?

Yes

Are sufficient details of methods and analysis provided to allow replication by others?

Yes

Reviewer Expertise:

Virtual Reality, Multimedia Learning, Computer-assisted Learning, Example-based Learning, Computer-supported Collaborative Learning, Productive Failure

I confirm that I have read this submission and believe that I have an appropriate level of expertise to confirm that it is of an acceptable scientific standard, however I have significant reservations, as outlined above.

## References

[1] : Using analysis of covariance (ANCOVA) with fallible covariates. *Psychol Methods* .2011;16(2) : 10.1037/a0023355 166-78 10.1037/a0023355 21517178

# Response

**Published:** 2022-12-23

**Authors:** Christian Fässler

**Affiliations:** ETH Zürich, Switzerland

*Dear Mr Hartmann! Thank you very much for your efforts and useful comments and suggestions!* 

1. Thank you for this useful hint! We omitted all abbreviations in the text except of “CVE”, “PS-I”, and “I-PS”. This is because these terms occur very frequently and we think is too cumbersome to read them out each time.
2. Thank you! We restructured the introduction and elaborated more on this topic for better clarifying this.
3. Thank you! We adapted accordingly and included a discussion about multimedia learning.
4. Thank you! We discussed on the PS-I fidelity criteria. However, we included more points for discussion to reflect more about respective PS-I mechanisms, but also feedback. Furthermore, we added a limitations section which might help for better understanding and clarification.
5. This makes sense, thank you! We added post-hoc statistical power values for each analysis.
6. In the text we were referring to the Extended Data where these figures could be found. However, now they are part of the main article.
7. Thank you! We added information about Cronbach’s alpha.
8. Thank you! Yes, this makes the text and discussion clearer. We added the stated research questions and related hypotheses. For deriving the hypotheses, we also added a whole section with past research on the topic of PS-I, problem-solving interventions in medical education, etc. Accordingly, we also included them specifically in our discussion.
9. Thank you! For better supporting our statement we added some sentences to describe our procedure. Furthermore, we provided an additional Extended Data file which illustrates the observed and imputed values for incomplete datasets. 10. Thank you! Please refer to our answer to the first comment of Mr. Lennart Schalk (reviewer 1).

# Peer review: Reviewer response for version 1

**DOI:** 10.21956/mep.20733.r32675

**Published:** 2022-11-01

**Authors:** Lennart Schalk

**Affiliations:** 1 Institute for Research on Instruction and Subject-Specific Education, PH Schwyz, Goldau, Switzerland

Generally, I believe this study comparing two sequences of learning with regard to the core disciplinary practice of differential diagnosis is well-described. It follows a strict and clear methodology, and the conclusions are mostly justified. However, I have one bigger issue (or maybe, it is rather a question for clarification or justification) with the statistical analyses and some minor issues that justify why I currently only approve the paper with reservations.

**Issue regarding the statistical analyses:**

 For most of the key hypotheses regarding learning gains, both repeated measures (rm) ANOVAs and ANCOVAs are conducted. What information does the rm ANOVA add? Please see Huck & McLean (1975) ^1^ for a discussion of rm ANOVAs vs. ANCOVAs vs. change score models. If an rm ANOVA is included in the results section, then the target effect would be the interaction (which is never reported). However, I see the ANCOVA to be more suitable for the present study, especially since the main effects of the rm ANOVA are not decisive. For example, the main effect of time (pre- vs. post-test) is difficult to interpret. It may reflect learning, but it may also simply reflect repeated testing (this possibility cannot be ruled out with the present design since there is no baseline condition without a learning intervention). So, if the rm ANOVA results remain included, then the effects of the interaction must be added. But it may be possible to shorten and simplify the results section by relying on ANCOVAs only. I would like the authors to consider these options and justify their plan for statistical analyses accordingly.

**Minor issues:**

 First, I think that dichotomizing transfer into near and far is an oversimplification (see p. 3). I suggest describing the transfer distance more precisely by utilizing the transfer dimensions described by Barnett and Ceci (2002) ^2^.

 Second, there is a sentence in the discussion (p. 10) starting with " *The lacking difference in triggered...*" which I do not understand. Please consider rewriting.

 Third, the authors should be a little bit more cautious in one part of the discussion. They write: " *Our findings indicate that only the CVE learning activity imparts CRS and that CRS cannot be taught by theoretical instruction only.*" (p. 10). Since there is no baseline, one cannot say CRS cannot at all be taught by the I part of the I-CVE sequence. Indeed, there are advantages for CVE-I, but they are only advantages and these advantages only emerge in specific CRS factors.

Have any limitations of the research been acknowledged?

Partly

Is the work clearly and accurately presented and does it cite the current literature?

Yes

If applicable, is the statistical analysis and its interpretation appropriate?

Partly

Are all the source data underlying the results available to ensure full reproducibility?

Yes

Is the study design appropriate and is the work technically sound?

Yes

Are the conclusions drawn adequately supported by the results?

Partly

Are sufficient details of methods and analysis provided to allow replication by others?

Yes

Reviewer Expertise:

Learning and instruction; cognitive psychology; STEM education

I confirm that I have read this submission and believe that I have an appropriate level of expertise to confirm that it is of an acceptable scientific standard, however I have significant reservations, as outlined above.

## References

[1] : Using a repeated measures ANOVA to analyze the data from a pretest-posttest design: A potentially confusing task. *Psychological Bulletin* .1975;82(4) : 10.1037/h0076767 511-518 10.1037/h0076767

[2] : When and where do we apply what we learn? A taxonomy for far transfer. *Psychol Bull* .2002;128(4) : 10.1037/0033-2909.128.4.612 612-37 10.1037/0033-2909.128.4.612 12081085

# Response

**Published:** 2022-12-23

**Authors:** Christian Fässler

**Affiliations:** ETH Zürich, Switzerland

*Dear Mr Schalk! Thank you very much for your efforts and useful comments and suggestions!*

**Issue regarding the statistical analyses:**

 Thank you very much for this useful comment! First, we included RM ANOVAs they are explicitly accounting for within-subject variability (separating it into signal and noise). Conversely, the regular between-subject ANOVA just treats all within-subject variability as error. Consequently, a repeated-measures ANOVA is making the signal stronger (by reducing overall noise). However, the second (and more relevant) reason why we used the RM ANOVA is that we were able to identify whether the learning activity sequences and the learning activities individually are effective in fostering learning at all (pre-post comparisons) and not only to compare the learning activity sequences with each other. One finding was possible only due to the application of the RM ANOVA was the issue was intermediate testing analysis from which we could draw that both learning activities are effective for learning declarative knowledge. This would not have been possible by only applying ANCOVAs. Even though this finding is not directly related to one of the research questions (which investigate differences between intervention groups), we think this is an insightful finding because most post-intervention comparisons remained non-significant. Nevertheless, we agree on relying to ANCOVAs only because there were no significant interaction effects in the RM ANOVAS, neither for intermediate, isomorphic, near transfer, nor far transfer declarative and conceptual clinical knowledge. Furthermore, and this is in alignment with comment 10 of the second reviewer, we ran new ANCOVA analyses where we excluded most covariates which were originally included and controlled for prior knowledge only. This is because, after all, all variables (except of prior knowledge) are part of the manipulation. Additionally, we reported statistical power for all analyses.

**Minor issues:**

1. Thank you very much for the detailed distinction of various distances of transfer provided in the stated reference! We added a whole section to elaborate on this.
2. Thank you for the hint! We adapted accordingly.
3. Thank you! We agree with your argumentation and adapted the respective sections accordingly.
